# Evidence for an early evolutionary emergence of γ-type carbonic anhydrases as components of mitochondrial respiratory complex I

**DOI:** 10.1186/1471-2148-10-176

**Published:** 2010-06-14

**Authors:** Ryan MR Gawryluk, Michael W Gray

**Affiliations:** 1 Centre for Comparative Genomics and Evolutionary Bioinformatics, Department of Biochemistry and Molecular Biology, Dalhousie University, Halifax, Nova Scotia B3H 1X5, Canada

## Abstract

**Background:**

The complexity of mitochondrial complex I (CI; NADH:ubiquinone oxidoreductase) has increased considerably relative to the homologous complex in bacteria. Comparative analyses of CI composition in animals, fungi and land plants/green algae suggest that novel components of mitochondrial CI include a set of 18 proteins common to all eukaryotes and a variable number of lineage-specific subunits. In plants and green algae, several purportedly plant-specific proteins homologous to γ-type carbonic anhydrases (γCA) have been identified as components of CI. However, relatively little is known about CI composition in the unicellular protists, the characterizations of which are essential to our understanding of CI evolution.

**Results:**

We have performed a tandem mass spectrometric characterization of CI from the amoeboid protozoon *Acanthamoeba castellanii*. Among the proteins identified were two γCA homologs, AcCa1 and AcCa2, demonstrating that γCA proteins are not specific to plants/green algae. In fact, through bioinformatics searches we detected γCA homologs in diverse protist lineages, and several of these homologs are predicted to possess N-terminal mitochondrial targeting peptides.

**Conclusions:**

The detection of γCAs in CI of *Acanthamoeba*, considered to be a closer relative of animals and fungi than plants, suggests that γCA proteins may have been an ancestral feature of mitochondrial CI, rather than a novel, plant-specific addition. This assertion is supported by the presence of genes encoding γCAs in the nuclear genomes of a wide variety of eukaryotes. Together, these findings emphasize the importance of a phylogenetically broad characterization of CI for elucidating CI evolution in eukaryotes.

## Background

Mitochondrial complex I (CI; NADH:ubiquinone oxidoreductase) is a multi-subunit and intricate proton pump that is responsible for the first step in the canonical respiratory chain - the oxidation of NADH and subsequent reduction of ubiquinone. Investigations of CI structure in animals and fungi portray CI as an L-shaped complex consisting of a hydrophobic domain integrated into the inner mitochondrial membrane and a hydrophilic mitochondrial matrix domain that oxidizes NADH and transfers electrons along 8-9 Fe-S clusters [1]. Mitochondrial CI comprises 35-45 proteins (~900-1000 kDa), which are encoded in both the mitochondrial and nuclear genomes. Conversely, the homologous ~550-kDa bacterial CI consists of only 14 subunits [2], suggesting a massive expansion of CI in eukaryotes.

The subunit composition of mitochondrial CI has been intensively studied in animals [3], fungi [4,5] and land plants/green algae [6,7]. Comparative proteomic and genomic analyses of CI composition in these groups have provided insight into the evolution of mitochondrial CI: specifically, in all three groups, the 14-subunit 'bacterial core' (corresponding to the subunits inherited from the α-proteobacterial ancestor of mitochondria; [8]) has been retained, while an additional 18-subunit 'eukaryotic core' (made up of proteins shared by these eukaryotic lineages but not by bacterial CI) along with a variable number of lineage-specific proteins has been added [9]. Few of the lineage-specific proteins are similar to other proteins of known function in available databases; however, in plants [7,10] and green algae [6], CI contains multiple proteins with high similarity to γ-type carbonic anhydrases (γCAs), first described as a homotrimeric complex (Cam) in the methanogenic archaeon, *Methanosarcina thermophila *[11].

Carbonic anhydrases are ubiquitous metalloenzymes that catalyze the reversible hydration of CO_2 _to HCO_3_^-^. Five evolutionarily unrelated CA classes (α, β, γ, δ, and ζ) are currently known, suggesting that this important enzymatic mechanism has been invented independently multiple times. The γCA class is among the most ancient, with homologs widespread in Archaea and Bacteria [12]; however, in eukaryotes, γCA homologs have been described from a phylogenetically narrow collection of species, comprising almost exclusively green algae and land plants, in association with CI. In this group, single-particle electron microscopy studies have demonstrated the presence of an additional CI domain [13,14]. This novel structure, likely representing the γCA proteins, is associated with the inner membrane portion of CI and projects into the mitochondrial matrix. The function of γCA proteins in CI remains enigmatic: thus far, carbonic anhydrase activity has not been detected biochemically. However, experiments in *Arabidopsis *have demonstrated that expression of plant γCAs is affected by CO_2 _concentration (see [15]) and that plant γCA proteins bind inorganic carbon [16]. These functional observations, coupled to the apparent restriction of γCAs to mitochondria of plant/green algal lineages, have prompted the proposal that CI γCA proteins may have plant-specific functions; for instance, Braun & Zabaleta [17] have suggested that γCA proteins play an important role in chloroplast function via the generation of HCO_3_^- ^in the recycling of inorganic carbon for CO_2 _fixation.

Although γCA proteins are not components of CI in animals and fungi (see [9]), little is known about the composition of CI in the predominantly unicellular protists. Because protists comprise the bulk of eukaryotic diversity, it is important to characterize CI from diverse lineages in order to understand fully the evolution of this protein complex. In particular, we cannot conclude that supposedly lineage-specific CI subunits are truly lineage-specific without knowledge of CI composition in the majority of eukaryotes. To this end, we have isolated an enzymatically active, ~940-kDa native CI and an inactive, ~820-kDa CI subcomplex (CI*) from the amoeboid protist *Acanthamoeba castellanii *via blue native polyacrylamide gel electrophoresis (BN-PAGE) and have carried out a characterization of subunit composition via tandem mass spectrometry (MS/MS). Here we describe the detection of two γCA homologs, AcCa1 and AcCa2, in both forms of *Acanthamoeba *CI. *Acanthamoeba *is a member of the eukaryotic supergroup Amoebozoa and is not a close relative of plants [18]; rather, available evidence indicates that Amoebozoa comprises the evolutionary sister group to the opisthokonts (animals, fungi and their specific unicellular relatives). The unexpected finding of γCA homologs in mitochondrial CI of *Acanthamoeba *demonstrates that these proteins are not limited to CI of plants/green algae, as was previously suggested [10]. In fact, we provide evidence here that γCA homologs having predicted mitochondrial targeting peptides (mTPs) are widespread throughout the domain Eucarya.

## Results and Discussion

### *γ Carbonic anhydrase proteins in *Acanthamoeba *CI*

**Figure 1:**
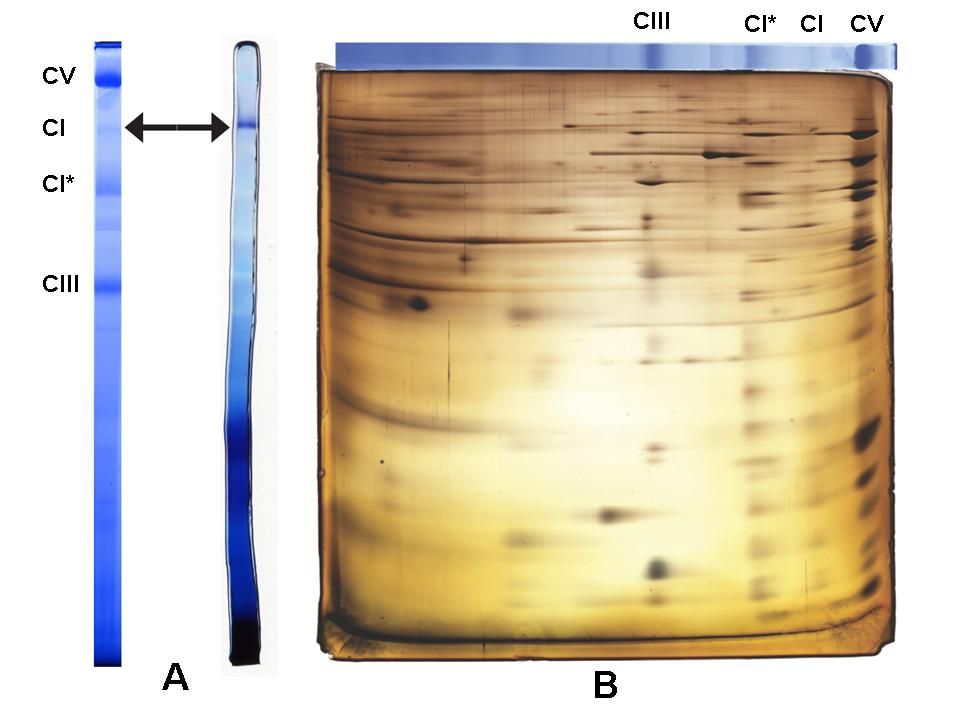
**BN-PAGE, CI enzyme activity (panel A) and two-dimensional BN/SDS-PAGE (panel B) profiles**. Panel A shows the characteristic Coomassie-stained BN-PAGE profile of *Acanthamoeba *mitochondrial complexes (left) and in-gel assay of these complexes (*Acanthamoeba *BN-PAGE lane from the same gel), identifying an NADH dehydrogenase activity at ~940 kDa (right). This band is considered to represent intact CI from *Acanthamoeba*. Panel B shows the protein profile observed after two-dimensional BN/SDS-PAGE of *Acanthamoeba *mitochondrial complexes resolved in panel A. A list of proteins identified in CI and CI* isolated from BN-PAGE bands is presented in Additional File 1. CI, intact complex I (940 kDa); CI* = CI subcomplex (820 kDa); CIII, complex III; CV, complex V.

An in-gel assay for NADH dehydrogenase activity was performed on *Acanthamoeba *mitochondrial protein complexes separated by BN-PAGE, yielding an insoluble formazan precipitate at ~940 kDa (Figure 1A). This molecular weight estimate is in agreement with the findings of Navet *et al. *[19] and corresponds to the general size of CI across eukaryotes. Together, these observations suggest that the identified NADH dehydrogenase activity corresponds to the native, enzymatically active CI of *Acanthamoeba*. Furthermore, analysis of *Acanthamoeba *mitochondrial protein complexes by two-dimensional BN/SDS-PAGE revealed another, more abundant complex (CI*) of ~820 kDa that has a very similar protein profile to intact CI (Figure 1B). Based on the two-dimensional BN/SDS-PAGE profile and lack of enzyme activity following one-dimensional BN-PAGE, we infer that this complex is likely a partially dissociated CI missing a portion of the NADH-oxidizing mitochondrial matrix domain. The dissociation of CI into subcomplexes during BN-PAGE is not uncommon, and the observed breakdown into a slightly smaller, enzymatically inactive subcomplex is reminiscent of the situation in the green alga *Polytomella *[14].

**Figure 2:**
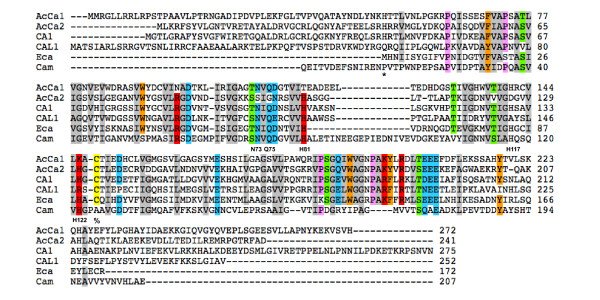
**Alignment of AcCa1 and AcCa2 sequences with homologs from *Arabidopsis *and prokaryotes**. *Acanthamoeba *AcCa1 and AcCa2 are aligned with *Arabidopsis *CA1 and CAL1 along with an α-proteobacterial (*Ehrlichia canis*) γCA homolog and archaeal Cam (*Methanosarcina thermophila*). Critical amino acids described in text are referred to according to the Cam nomenclature. H42 and C137 of CA1 are denoted by * and %, respectively. Shading of columns reflects amino acid similarity of ≥ 80%.

Tandem mass spectrometric analysis of the 940-kDa and 820-kDa bands demonstrated that both were enriched in CI subunits known from other organisms, supporting our conclusion that both represent CI (see Additional File 1). A number of contaminating, high-abundance mitochondrial proteins from other complexes were also present; this is not unexpected, as the relative abundance of CI is low in comparison with other complexes, and CI* appears to co-migrate with other abundant complexes (Figure 1B). Notably, two γCA homologs were detected in both the 940-kDa and 820-kDa samples. These proteins, here named AcCa1 and AcCa2, are 36% identical to each other and are the only γCA homologs represented in the *Acanthamoeba *TBestDB EST library [20] and the ongoing *Acanthamoeba *nuclear genome project (Baylor College of Medicine; http://www.hgsc.bcm.tmc.edu/microbial-detail.xsp?project_id=163). Between the two samples analyzed by MS/MS, 31% and 47% of the AcCa1 and AcCa2 protein sequences were covered, respectively (see Additional File 2). These findings constitute the first report of γCA homologs as CI components outside of the plant/green algal lineage (see Figure 2 for multiple alignment). Based on these results, we suggest that AcCa1 and AcCa2 are matrix-facing components of the inner membrane arm of CI in *Acanthamoeba*, as are their homologs in plants/green algae [13,14].

Because not all expected CI subunits were detected in the MS/MS analysis and because the main contaminants of our CI proteins were ATP synthase proteins, we considered the possibility that the γCA proteins might actually be ATP synthase (CV) subunits. The enzymatically active CI complex is present in very low abundance in *A. castellanii*, so that detection of all CI subunits is proving to be a challenge. Conversely, CV is extraordinarily abundant, as is evident in Figures 1a and 1b; as a result, MS/MS data have revealed all CV subunits in *A. castellanii*, whereas no γCA proteins were detected in this complex. These considerations combined with the fact that γCA proteins have been characterized as *bona fide *CI components in other organisms effectively eliminates the possibility that they might be CV subunits instead in *Acanthamoeba*.

A small number of γCA homologs have been described in eukaryotes outside of the plant supergroup. A cDNA encoding a γCA homolog from the haptophyte alga *Emiliania huxleyi *was identified previously [21]; however the authors did not investigate the subcellular localization of the protein. We have previously identified three different γCA homologs in a proteomic investigation of highly purified mitochondria from the cilated protozoon, *Tetrahymena thermophila *[22]. Interestingly, two of these three γCA homologs were detected in mass spectrometric analyses of a 1 MDa aggregate from *Tetrahymena *mitochondrial membrane proteins separated by BN-PAGE (Gawryluk *et al*., unpublished results). This aggregate is highly enriched in known protein components of complexes I, III and V, suggesting that the identified γCA homologs may be components of CI in *Tetrahymena *as well.

From an evolutionary perspective, our findings are somewhat surprising: the Amoebozoa supergroup (to which *Acanthamoeba *belongs) is thought to be sister to the opisthokonts (animals + fungi), and is not closely related to the Plantae supergroup [18]. Since no authentic γCA homologs were detected in opisthokont nuclear genome or EST sequence databases (and no γCA homologs are part of opisthokont CI), these results suggest that γCA proteins may have been specifically lost from opisthokont CI, rather than recently added to the plant/green algal lineage. Moreover, the finding that γCA homologs may be components of CI in other eukaryotic groups (ciliates) raises the possibility that γCA homologs were an ancestral feature of mitochondrial CI (i.e., a component of the eukaryotic core of CI lost in animals and fungi). We expected that if this were the case, γCA homologs should be detected in other protist lineages.

### γ Carbonic anhydrase proteins are found throughout Eucarya

In order to better understand the distribution and evolution of γCA homologs across the domain Eucarya, we employed a systematic bioinformatics approach to search nuclear genomic and EST sequence databases from diverse eukaryotes. In contrast to previous reports that γCA proteins are largely limited to plants/green algae, we detected (often multiple) homologs in the majority of eukaryotic groups (Table 1; see also Additional Files 3, 4 and 5). γCA homologs are found in other major groups within the Plantae supergroup, including one in the red alga *Cyanidioschyzon merolae *and at least two in the glaucophyte alga, *Cyanophora paradoxa*. Aside from the opisthokonts, homologs could be detected in most eukaryotic supergroups, including Amoebozoa (*Acanthamoeba*, *Dictyostelium*, *Hartmannella, Hyperamoeba*, *Polysphondylium*), Chromalveolata (*Tetrahymena*, *Phytophthora*, *Phaeodactylum*, *Blastocystis*, *Emiliania*, *Guillardia*), Excavata (*Reclinomonas*, *Seculamonas*, *Naegleria*, *Euglena*, *Trypanosoma*, *Malawimonas*) and possibly Rhizaria (an EST homolog was idenitified in *Bigelowiella natans*; however, several stop codons interrupt the reading frame, suggesting that the EST in question may represent an incompletely spliced transcript).

**Table 1:** Distribution of γCAs Throughout Eukaryotes.

Supergroup	Taxon	γCA homolog ?	Number of γCA homologs	
Plantae	*Arabidopsis*	Yes	5	All are CI subunits

	*Chlamydomonas*	Yes	3	All are CI subunits

	*Cyanidioschyzon*	Yes	1	

	*Cyanophora*	Yes	≥ 2	

Amoebozoa	*Acanthamoeba*	Yes	2	Both are CI subunits

	*Dictyostelium*	Yes	2	

	*Hartmannella*	Yes	≥ 1	

	*Entamoeba*	Yes	1	No ETC

	*Polysphondylium*	Yes	2	

Opisthokonta	*Saccharomyces*	No	0	

	*Neurospora*	No	0	

	*Ustilago*	No	0	

	*Yarrowia*	No	0	

	*Bos*	No	0	

	*Drosophila*	No	0	

	*Monosiga*	No	0	

Chromalveolata	*Tetrahymena*	Yes	3	All are mitochondrial

	*Plasmodium*	No	0	Lacks CI

	*Phytophthora*	Yes	2	

	*Phaeodactylum*	Yes	≥ 2	

	*Blastocystis*	Yes	≥ 1	

	*Guillardia*	Yes	≥ 3	

	*Emiliania*	Yes	≥ 1	

Rhizaria	*Bigelowiella*	Yes	≥ 1	

Excavata	*Reclinomonas*	Yes	≥ 1	

	*Naegleria*	Yes	2	

	*Euglena*	Yes	≥ 1	

	*Trypanosoma*	Yes	2	

	*Malawimonas*	Yes	≥ 2	

	*Trichomonas*	No	0	Lacks ETC

	*Giardia*	No	0	Lacks ETC

Organisms are arranged according to supergroup membership [18]. 'Number of γCA homologs' refers to the number of distinct γCA proteins encoded by an organism as determined by searches of EST or nuclear genomic databases. Inferred protein sequences are available in Additional Files 3 and 4

Of the retrieved γCA protein sequences considered to be complete at their N-termini (where mTPs would be located; [23]), many are predicted to be mitochondrial by the programs TargetP [24] and MitoProtII [25] above a confidence level of 70%, suggesting that γCA proteins may be targeted to mitochondria in a wide variety of eukaryotes (see Additional File 6). Notably, several of the known mitochondrial γCA proteins are not predicted to have mTPs with a given program (e.g. CA3 and CAL2 from *Arabidopsis*, AcCa2 from *Acanthamoeba *and all three γCAs from *Tetrahymena *with TargetP), so the lack of an apparent mTP does not preclude a mitochondrial localization. Genes encoding γCA proteins are absent from the nuclear genome sequences of several eukaryotes that do not have conventional mitochondria (e.g. *Trichomonas*, *Giardia*) and are also absent from at least one eukaryote (*Plasmodium*) that specifically lacks CI, further suggesting that γCA proteins may be targeted to mitochondria - and likely to CI - in most eukaryotes. Surprisingly, a γCA homolog was detected in the nuclear genome of *Entamoeba histolytica *[26], an amoebozoon that does not possess conventional mitochondria (i.e., it lacks an electron transport chain). This homolog, however, does not appear to be closely related to other eukaryotic γCAs, and may represent a lateral gene transfer from bacteria (data not shown).

To assess evolutionary relationships, we performed a phylogenetic analysis of eukaryotic and bacterial γCA proteins, using the archaeal Cam sequence as outgroup. In the resulting phylogenetic trees (not shown), virtually all eukaryotic γCA proteins formed a large clade branching as a sister group to α-Proteobacteria (the *B. natans *sequence branched within the latter clade whereas the *E. histolytica *sequence mentioned above, as well as a *G. theta *one, did not affiliate with the other eukaryotic γCA sequences, but neither did they branch robustly with any particular bacterial clade). However, these trees were poorly resolved, with very little statistical (bootstrap) support for most partitions, so the phylogenetic conclusions that can be drawn from them are limited. Although the available data (Table 1) suggest an early γCA gene duplication during eukaryotic evolution, phylogenetic analysis was not able to delineate the point at which this may have occurred. We can infer, however, that the duplication predated the divergence of the amoebozoan taxa investigated here; moreover, we are able to identify the amoebozoan sequences that are orthologous with either AcCa1 or AcCa2 (Additional File 5).

### Analysis of primary protein structure of γCA homologs

As earlier noted, plant and green algal γCA proteins constitute a CI domain that is associated with the matrix face of the inner membrane-integrated arm [13,14]. *Arabidopsis *encodes five γCA homologs (CA1, CA2, CA3, CAL1 and CAL2), all of which interact with CI [27], while *Chlamydomonas *CI contains three different γCA homologs [6]. Although *Arabidopsis *γCA1-3 (but not CAL1, CAL2) have retained nearly all residues known to be structurally or functionally important in the archetypal γCA (Cam) from the methanogenic archaeon *Methanosarcina thermophila *[11], including the Zn^2+^- binding residues H81, H117 and H122 [10], the function of γCA homologs in plant mitochondria is still largely unknown. Plant γCA proteins lack two Glu residues (D61, D84) critical to the proton transfer mechanism of Cam and all previous attempts to demonstrate carbonic anhydrase activity in *Arabidopsis *mitochondrial extracts and in sucrose gradient-enriched plant CI [15] and recombinant γCA2 homotrimers [16] have been unsuccessful. Like the plant homologs, *Acanthamoeba *γCAs lack D61 and D84; moreover, *Acanthamoeba *γCAs conserve fewer of the other residues critical to Cam function than do plant γCAs. In terms of Zn^2+^-binding His residues, AcCa1 conserves only H117 whereas AcCa2 retains H81 and H122 (Figure 2). However, it should be noted that the three His residues required to bind Zn^2+ ^in Cam are not localized within the same γCA monomer: rather, Zn^2+ ^is coordinated by H81 and H122 of one monomer and H117 of a second monomer within the trimeric Cam structure [28]. It is noteworthy that the three His residues are distributed between the two γCA homologs found in *Acanthamoeba*, so it may be that the latter are able to bind Zn^2+ ^in concert. However, in AcCa2, S and G replace N73 and Q75, respectively; in Cam, N73 and Q75 are proposed to be critical components of the catalytic mechanism [28]. Taken together, these findings suggest that the γCA homologs of *Acanthamoeba *CI may not possess a carbonic anhydrase activity. On the other hand, carbonic anhydrase activity has been reported for a comparably divergent, recombinant γCA from the haptophyte *Emiliania huxleyi *[21].

As is the case with the *Acanthamoeba *proteins, the vast majority of γCA proteins from eukaryotes outside of the plant supergroup do not individually conserve all three metal-binding His residues, although all three are often found among different γCAs where multiple isoforms are present. Thus, it is still unclear whether carbonic anhydrase activity or Zn^2+ ^binding is likely to be a general feature of γCAs across eukaryotes. Interestingly, several other positions in our phylogenetically broad multiple alignment are fairly well conserved across eukaryotes (and in some cases, bacteria), but not in Cam; it is possible that the conservation of these residues points to novel, potentially important functional sites. For instance, a His residue (corresponding to H42 of *Arabidopsis *γCA1) is present in 27 of the 40 eukaryotic homologs with complete N-terminal sequence that we identified in this study, but not in bacterial/archaeal γCA homologs, while a Cys residue (*Arabidopsis *γCA1 C137) is highly conserved in eukaryotes and bacteria (41/48), but not in Cam (see Additional File 4). Additionally, many of the eukaryotic γCA homologs (including the *Acanthamoeba *ones) have C-terminal extensions relative to their prokaryotic homologs. In land plants, these extensions have been implicated in integration into the inner mitochondrial membrane [14], further supporting the idea that γCA proteins may be components of CI in many diverse eukaryotes.

**Figure 3:**
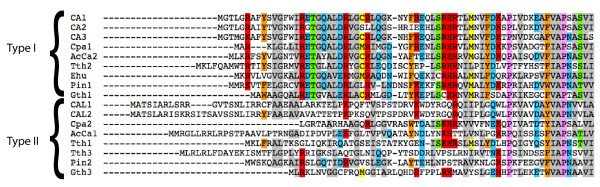
**Distinct subtypes of eukaryotic γCAs**. A truncated alignment of γCA proteins demonstrates that the N-terminal regions of certain isoforms (termed Class I) are highly conserved throughout Eucarya (see text). Species abbreviations: CA1-3, *Arabidopsis*; Cpa, *Cyanophora paradoxa*; AcCa, *Acanthamoeba castellanii*; Tth, *Tetrahymena thermophila*; Ehu, *Emiliania huxleyi*; Pin, *Phytophthora infestans*; Gth, *Guillardia theta*. Shading of columns reflects amino acid similarity of ≥ 40%.

Interestingly, inspection of the N-terminal portion of γCAs from diverse eukaryotes demonstrates that there may be, in general, at least two distinct classes of γCA proteins. In particular, the N-terminal ~60 amino acids of AcCa2, along with various protist γCAs, are highly similar to the corresponding region of *Arabidopsis *CA1-3, whereas the N-termini of AcCa1 and *Arabidopsis *CAL1-2 are not highly conserved (Figure 3). This observation suggests that there may be distinct roles or sub-localizations of the two classes of γCA within CI; specifically, the high degree of conservation of the N-terminal region of the AcCa2/CA1-3 class, but not AcCa1/CAL1-2 class, implies that the functional constraints at protein N-termini are stronger in the former class.

### Possible function(s) of γCA proteins in CI

Although *in silico *reconstructions of *Arabidopsis *γCA proteins and comparisons to Cam suggest the possibility of carbonic anhydrase activity, the biochemical function of γCA homologs in plant/green algal CI is controversial as there are currently no experimental data confirming that plant/green algal γCA proteins are carbonic anhydrases. Recently, however, it has been shown that *Arabidopsis *γCA2 trimers are capable of binding inorganic carbon [16], and microarray studies indicate that expression of *Arabidopsis *γCA1 and γCA2 is down-regulated under high CO_2 _concentrations (see [15]). These results suggest that γCA homologs might play an important role in the metabolism or transport of one-carbon compounds in land plants. Although these observations provide evidence that γCAs function in relation to inorganic carbon metabolism in plants, it is still unclear whether these observations can be generalized across eukaryotes. As discussed above, most eukaryotes do not conserve all three His residues required for Zn^2+ ^coordination (and therefore binding of CO_2 _and HCO_3_^-^). Consequently, it may be that the function of γCA proteins is somewhat different in plants and other eukaryotes, and that CI γCA proteins in non-plant species serve a predominantly structural role in the complex. Ultimately, in order to distinguish between these possibilities, it will be important to determine whether or not non-plant γCA proteins are capable of binding Zn^2+^.

Because CI γCA proteins were believed to be plant-specific, it has been proposed that they are involved in plant-specific processes, namely HCO_3_^- ^formation (and possibly HCO_3_^- ^transport across the inner mitochondrial membrane), ultimately for CO_2 _fixation in chloroplasts [17]. Although we cannot rule out the possibility that CI γCAs play an active role in chloroplast metabolism in plants, the presence of γCA homologs in CI of ancestrally aplastidic eukaryotes (e.g. *Acanthamoeba*) demonstrates that this could not have been the ancestral role of γCAs in CI, and that these proteins must perform some role that is not related to chloroplast function. Other data suggest that γCA proteins in plants may play a role in expression and assembly of mitochondrial CI. For instance, it was demonstrated that total levels of CI, along with abundance of CI+CIII supercomplexes, are dramatically decreased (>80%) in *Arabidopsis *γCA2 and γCA3 knockout lines grown in suspension culture [15]. A reduction in total CI protein was detected (i.e., not just aberrant CI assembly), suggesting that γCA proteins play an important role in the expression/stability of CI subunits. Recently, Tripp *et al. *[29] demonstrated that the active site of Cam contains Fe^2+ ^instead of Zn^2+ ^when the enzyme is reconstituted under anaerobic conditions. As noted by Parisi *et al. *[10], CI (along with the rest of the respiratory chain) contains multiple iron centers, so the Fe-binding ability of Cam may point to a role for γCA proteins in mitochondrial respiratory control. Notably, *Arabidopsis *γCA2, a physical component of CI, has been annotated as a transcription factor involved in the anther-specific expression of nucleus-encoded CI proteins (see GenBank accession no. AAK28403). The prospect that γCA proteins may function as CI-specific transcription factors is quite intriguing, as it would suggest a novel mechanism of communication between the mitochondrial electron transport chain and the nucleus, and would indicate a function for CI γCA proteins that could be applicable across all aerobic CI-containing eukaryotes, and not just photosynthetic ones.

## Conclusions

The number of subunits comprising CI in eukaryotes has expanded markedly relative to the homologous bacterial complex. Previous comparative genomic analyses have suggested that 18 proteins were added to CI very early in eukaryotic evolution, while a smaller proportion are specific to particular lineages within the eukaryotic domain [9]. Although a number of the novel, 'lineage-specific' proteins are likely to be encoded by a phylogenetically restricted group of related organisms, others are currently deemed to be 'lineage-specific' only because our knowledge of CI composition across the eukaryotes is incomplete. We have detected two γCA homologs - previously thought to be plant/green algal CI proteins - as components of CI in an amoeboid protozoon, *Acanthamoeba castellanii*, an organism not specifically related to the plant/green algal lineage. The most parsimonious interpretation of our observations is that γCA proteins were part of the eukaryotic core of CI that was added early in eukaryotic evolution, and that they were subsequently lost in certain groups, most notably the opisthokonts. Moreover, γCA proteins are known to be mitochondrial in ciliates (and may be CI proteins as well) and bioinformatics searches have revealed a large repertoire of γCA homologs in other major eukaryotic groups. These observations underscore the importance of characterizing the composition of mitochondrial protein complexes from a wide variety of organisms in order to understand fully and accurately their function and evolution.

## Methods

### Cell Growth and Isolation of Mitochondria

Mitochondria were prepared from two 500-ml cultures of *Acanthamoeba castellanii *(strain Neff) cells grown to an OD_580 _of ~1.0, essentially as reported by Lohan and Gray [30]. The present method differed in that cells were not washed with phosphate-buffered saline prior to lysis, the crude mitochondrial pellet was washed only once with mitochondrial wash buffer (10 mM Tris·HCl, 10 mM Na_2_EDTA, 0.35 M sucrose, 1 mM dithiothreitol and 0.1% BSA) and mitochondria were purified in a SW27Ti rotor centrifuged at 22,000 rpm. The protein concentration was assayed with the BioRad *D_C _*Protein Assay kit and whole mitochondria were diluted to a final protein concentration of 25 mg/ml.

### Blue Native Polyacrylamide Gel Electrophoresis (BN-PAGE)

BN-PAGE was carried out according to Schägger and von Jagow [31]. A 4-μl aliquot of purified mitochondrial fraction (corresponding to 100 μg protein) was solubilized with 40 μl of a solution containing 0.5% n-dodecyl-β-D-maltoside in 750 mM 6-aminocaproic acid/50 mM bis-tris [bis-(2-hydroxyethyl)-amino-tris(hydroxymethyl)-methane] for 15 min on ice. The solution was centrifuged at 18,000 × *g *for 30 min at 4°C in order to sediment insoluble material. The supernatant was supplemented with 1.5 μl 5% Coomassie Brilliant Blue G250 in 750 mM 6-aminocaproic acid/50 mM bis-tris and 5.0 μl 50% (v:v) glycerol. Samples were loaded onto a 1.5-mm thick, 4-12% polyacrylamide linear gradient gel without a stacking gel and electrophoresed for 2 hr at 150 V, 2 hr at 350 V and ~5 hr at 500 V at 4°C. Gels were either a) stained with a solution of 0.025% Coomassie Brilliant Blue R250, 40% ethanol, 10% acetic acid or b) gel lanes were excised for in-gel enzyme activity assays or two-dimensional BN/SDS-PAGE. Molecular weights were estimated using the NativeMark unstained protein standard (Invitrogen).

### In-gel Enzyme Activity Assay

In order to identify enzymatically active CI, in-gel activity assays were performed. A lane from a BN-PAGE experiment was excised and incubated overnight in 5 ml 50 mM MOPS·NaOH, pH 7.4 buffer containing 0.1 mg/ml reduced β-NADH and 1 mg/ml nitroblue tetrazolium. Active CI was identified by the formation of a purple-blue formazan precipitate at approximately 940 kDa.

### Two-dimensional BN/SDS-PAGE

After one-dimensional BN-PAGE, a gel strip was excised and treated for 45 min with 10 ml of a 0.125 M Tris·HCl buffer, pH 6.8 (1× SDS-PAGE stacking buffer), containing 2% sodium dodecyl sulfate (SDS) and 1% β-mercaptoethanol. The strip was incubated for 10 min in 10 ml of the same solution, minus β-mercaptoethanol (a potent inhibitor of polymerization). The BN gel strip was encased in 4% SDS-PAGE stacking gel poured on top of a 15% resolving gel. Electrophoresis was carried out at room temperature at a constant current of 30 mA for approximately 6 hr. The gel was stained with the BioRad Silver Stain Plus kit according to manufacturer's protocols.

### Reversed-phase HPLC and MS/MS

The enzymatically active CI and inactive CI complexes were excised and each was placed in a separate microcentrifuge tube. The proteins were reduced with 10 mM dithiothreitol in 100 mM ammonium bicarbonate (AB) for 30 min at 56°C and alkylated with 100 mM iodoacetamide in 100 mM AB for 30 min in the dark at room temperature. Proteins were incubated with trypsin overnight (~16 hr) at 37°C with 12.5 ng/μl modified porcine trypsin (Promega) in 100 mM AB. Peptides were extracted once with 100 mM AB and twice with a 50:50 (v:v) acetonitrile:H_2_O solution containing 0.2% formic acid. All samples were analyzed by LC-MS/MS using an Agilent 1100 HPLC system equipped with a 15 cm × 100 mm Onyx Monolythic C_18 _column (Phenomenex, Torrance, California). The separation was carried out using the following gradient: 2% B for 3 min increasing to 25% B over 45 min and 95% B over 10 min (A: 0.1% formic acid in water; B: 0.1% formic acid in acetonitrile) at 2 μl/min. The HPLC was interfaced to an AB/MDS-SCIEX QTrap 4000 mass spectrometer via a nanoflow source. Data were acquired in the information-dependent acquisition mode, i.e., the m/z values of the tryptic peptides were measured using an MS scan, followed by enhanced resolution scans of the three most intense peaks and finally three tandem MS scans. The tandem MS spectra were submitted to the database search program MASCOT [32] in order to identify the proteins. Data files were searched against *A. castellanii *EST clusters from TBestDB [20], a collection of *Acanthamoeba *ESTs generated by 454 pyrosequencing (kindly made available by BJ Loftus, University College Dublin), the current nuclear genome assembly (*A. castellanii *genome project), and the *A. castellanii *mitochondrial genome sequence [33]. The current *A. castellanii *genome assembly and Loftus EST data set are both publicly available from the Human Genome Sequencing Center, Baylor College of Medicine http://www.hgsc.bcm.tmc.edu/projects/microbial/microbial-pubPreview.xsp?project_id=163.

### Homology searches, alignments, mTP prediction

The amino acid sequence of *Arabidopsis thaliana *γCA1 (AT1G5980) was used to query the nr protein database at NCBI with BLASTp, as well as EST and nuclear genome databases from a wide variety of eukaryotes with tBLASTn [34]. Nucleic acid sequences were translated using the transeq program of the EMBOSS package and protein sequences were inferred manually. Protein homologs were aligned with Muscle v3.6 [35] using default parameters and edited with the BioEdit Sequence Alignment Editor.

TargetP [24] and MitoProt II [25] were used to assess the probability of mitochondrial localization for eukaryotic γCA homologs. When TargetP was used, the 'Plant' organism group was selected for organisms with primary or secondary plastids in order to include the possibility of plastid-targeted γCA proteins; for species lacking a plastid, the 'Animal' organism group was selected. MitoProt II does not have an option for assessing plastid localization.

### Phylogenetic analysis

The AcCa1 protein sequence was used to query the nr database at NCBI via BLASTp. The best scoring homologs (i.e., ones having the lowest E- values) from a variety of major bacterial groups were retrieved and aligned with eukaryotic γ-type CA homologs using Muscle v.3.6 [35]. The alignment was edited manually with eBioX and was then used to reconstruct the maximum likelihood phylogenetic tree using RAxML-HPC [36] under the WAG + Γ model (using the PROTGAMMAWAGF option) with 25 categories of substitution rate variation. One hundred bootstrap replicates were performed as a measure of statistical support for inferred nodes.

## List of abbreviations

CI: complex I of the respiratory chain; γCA: γ-type carbonic anhydrase; Cam: archetypal γCA from *Methanosarcina thermophila*; AcCa1 and AcCa2: names given to γ-type carbonic anhydrases from *Acanthamoeba*; NADH: nicotinamide adenine dinucleotide (reduced); BN-PAGE: blue native polyacrylamide gel electrophoresis; mTP: mitochondrial targeting peptide; EST: expressed sequence tag; ETC: electron transport chain

## Authors' contributions

RMRG performed the mitochondrial isolations, BN-PAGE, two-dimensional BN/SDS-PAGE and sample preparation for MS/MS. RMRG performed bioinformatic analyses. RMRG and MWG prepared the manuscript. Both authors read and approved the final manuscript.

## Supplementary Material

Additional File 1 Table of CI and CI* proteins identified by tandem mass spectrometry. The table lists proteins identified in the 940 kDa (CI) and 820 kDa (CI*) complexes via MASCOT searches of a six-frame translation of a 454 EST database (N) and predicted proteins from the *Acanthamoeba *mitochondrial genome (M). Proteins with ion scores of 37 and above are reported from the 454 EST database while ion scores of 29 or higher are reported for inferred mtDNA-encoded proteins. Bolded entries highlighted in color indicate that the protein in question was identified in both CI and CI* complexes. The two categories designated 'Other Proteins' are assumed to be contaminants; in particular, CV components (ATP synthase subunits) appear to be exceptionally abundant mitochondrial proteins, as judged by the results of MS/MS analysis of whole mitochondria. Click here for file

Additional File 2 **MS/MS peptide coverage**. The peptides identified by MS/MS analysis in AcCa1 and AcCa2 are mapped onto the corresponding protein sequences. Each peptide is colour-coded according to whether it was identified only in the 940 kDa CI, only the 820 kDa CI*, or in both samples. Click here for file

Additional File 3 **Sequences of γCA proteins**. The inferred protein sequences of γCA proteins are reported in fasta format. Proteins are designated as indicated in Additional File 5. Click here for file

Additional File 4 **Phylogenetically broad alignment of eukaryotic and prokaryotic γCAs**.: A multiple alignment of eukaryotic and prokaryotic γCAs including complete and partial sequences from diverse eukaryotic groups. Click here for file

Additional File 5 **Table of accession numbers**. The table compiles accession numbers and other relevant information for γCA homologs available in public databases. Click here for file

Additional File 6 **Table of mitochondrial targeting probabilities**. The table reports the probabilities of mitochondrial localization for γCA proteins (according to TargetP and MitoProtII). Bolded entries indicate proteins predicted to be mitochondrial with ≥ 70% probability. Click here for file
