# Supplementary material for: Evidence for an early evolutionary emergence of γ-type carbonic anhydrases as components of mitochondrial respiratory complex I
Source: BMC Evol Biol. 2010 Jun 14;10:176. doi: 10.1186/1471-2148-10-176 (PMC2900272; doi:10.1186/1471-2148-10-176)
Supplement: Additional File 2 — MS/MS peptide coverage. The peptides identified by MS/MS analysis in AcCa1 and AcCa2 are mapped onto the corresponding protein sequences. Each peptide is colour-coded according to whether it was identified only in the 940 kDa CI, only the 820 kDa CI*, or in both samples. [file 1471-2148-10-176-S2.DOC]

**Additional File 2: MS/MS peptide coverage**

**Red** highlight refers to tryptic peptides identified in 940 kDa complex (CI)

**Blue** highlight refers to tryptic peptides identified in 820 kDa complex (CI*)

**Orange** highlight refers to tryptic peptides identified in both CI and CI*

**>AcCa1 – predicted 29.8 kDa, pI = 5.45, 31% coverage, combined ion score = 283**

MMRGLLRRLRPSTPAAVLPTRNGADIPDVPLEKFGLTVPVQATAYNDLYNK**HTTLVNLPGK**RPQISSESFVAPSATLVGNVEVWDR**ASVWYDCVINADTK**LIRIGAGTNVQDGTVITEADEELTEDHDGSTIVGHWVTIGHRCVLKACTIEDHCLVGMGSVLGAGSYMESHSILGAGSVLPAWQR**IPSGQIWVGNPAK**YLR**DLTEEEFDFLEKSSAHYTVLSK**QHAYEFYLPGHAYIDAEK**KGIQVGYQVEPLSGEESVLLAPNYK**EKVSVH*

**>AcCa2 – predicted 26.1 kDa, pI = 6.00, 47% coverage, combined ion score= 492**

MLKRFSYVLGNTVRETAYALDRVGCR**LQGNYAFTEELSR**HRRVMGLYDKQPAISQDVFIAPNASVIGSVSLGEGANVWYGSVLR**GDVNDISVGK**KSSIGNR**SVVHASGGLTTLAPTKIGDNVVVGDGVVLHGCTLEDECRVDDGAVLNDNVVVEKHAIVGPGAVVTSGK**R**VPSGQVWAGNPAK**YVRDVSEEEK**EFAGWAEK**RYTQAKAHLAQTIKLAEEKEVDLLTEDILREMRPGTRFAD*
