# Supplementary material for: Evidence for an early evolutionary emergence of γ-type carbonic anhydrases as components of mitochondrial respiratory complex I
Source: BMC Evol Biol. 2010 Jun 14;10:176. doi: 10.1186/1471-2148-10-176 (PMC2900272; doi:10.1186/1471-2148-10-176)
Supplement: Additional File 3 — Sequences of γCA proteins. The inferred protein sequences of γCA proteins are reported in fasta format. Proteins are designated as indicated in Additional File 5. [file 1471-2148-10-176-S3.DOC]

**Additional File 3: Sequences of γCA proteins**

>CA1_Arabidopsis_thaliana

MGTLGRAFYSVGFWIRETGQALDRLGCRLQGKNYFREQLSRHRTLMNVFDKAPIVDKEAF

VAPSASVIGDVHIGRGSSIWYGCVLRGDVNTVSVGSGTNIQDNSLVHVAKSNLSGKVHPT

IIGDNVTIGHSAVLHGCTVEDETFIGMGATLLDGVVVEKHGMVAAGALVRQNTRIPSGEV

WGGNPARFLRKLTDEEIAFISQSATNYSNLAQAHAAENAKPLNVIEFEKVLRKKHALKDE

EYDSMLGIVRETPPELNLPNNILPDKETKRPSNVN

>CA2_Arabidopsis_thaliana

MGTLGRAIYTVGNWIRGTGQALDRVGSLLQGSHRIEEHLSRHRTLMNVFDKSPLVDKDVF

VAPSASVIGDVQIGKGSSIWYGCVLRGDVNNISVGSGTNIQDNTLVHVAKTNISGKVLPT

LIGDNVTVGHSAVIHGCTVEDDAFVGMGATLLDGVVVEKHAMVAAGSLVKQNTRIPSGEV

WGGNPAKFMRKLTDEEIVYISQSAKNYINLAQIHASENSKSFEQIEVERALRKKYARKDE

DYDSMLGITRETPPELILPDNVLPGGKPVAKVPSTQYF

>CA3_Arabidopsis_thaliana

MGTMGKAFYSVGFWIRETGQALDRLGCRLQGKNHFREQLSRHRTLMNVFDKTPNVDKGAF

VAPNASLSGDVHVGRGSSIWYGCVLRGDANSISVGAGTNIQDNALVHVAKTNLSGKVLPT

VIGDNVTIGHSAVLHGCTVEDEAYIGTSATVLDGAHVEKHAMVASGALVRQNTRIPSGEV

WGGNPAKFLRKVTEEERVFFSSSAVEYSNLAQAHATENAKNLDEAEFKKLLNKKNARDTE

YDSVLDDLTLPENVPKAA

>CAL1_Arabidopsis_thaliana

MATSIARLSRRGVTSNLIRRCFAAEAALARKTELPKPQFTVSPSTDRVKWDYRGQRQIIP

LGQWLPKVAVDAYVAPNVVLAGQVTVWDGSSVWNGAVLRGDLNKITVGFCSNVQERCVVH

AAWSSPTGLPAATIIDRYVTVGAYSLLRSCTIEPECIIGQHSILMEGSLVETRSILEAGS

VVPPGRRIPSGELWGGNPARFIRTLTNEETLEIPKLAVAINHLSGDYFSEFLPYSTVYLE

VEKFKKSLGIAV

>CAL2_Arabidopsis_thaliana

MATSLARISKRSITSAVSSNLIRRYFAAEAVAVATTETPKPKSQVTPSPDRVKWDYRGQR

QIIPLGQWLPKVAVDAYVAPNVVLAGQVTVWDGSSVWNGAVLRGDLNKITVGFCSNVQER

CVVHAAWSSPTGLPAQTLIDRYVTVGAYSLLRSCTIEPECIIGQHSILMEGSLVETRSIL

EAGSVLPPGRRIPSGELWGGNPARFIRTLTNEETLEIPKLAVAINHLSGDYFSEFLPYST

IYLEVEKFKKSLGIAI

>Cre1_Chlamydomonas_reinhardtii

MSLFKSSLPAGFLFPYRHPKAKGLVEGTLYGLGSLFRGVGAALDELGSMVQGPQGSVKDH

VQPNLAFAPVHRKPDVPVNAGQVVPAPPAAARTLKIKEVVVPNKHSTAFVAANANVLGNV

KLGAGSSVWYGAVLRGDVNGIEVGANSNIQDNAIVHVSKYSMDGTARPTVIGNNVTIGHA

ATVHACTIEDNCLVGMGATVLDGATVKSGSIVAAGAVVPPNTTIPSGQVWAGSPAKFLRH

LEPEEASFIGKSASCYAELSAIHKFEQSKTFEEQYTESCIIKDRAALADPSNSVHQMWEY

DSQTALVARAKR

>Cre2_Chlamydomonas_reinhardtii

MNPINGLKTILHRVGFAMRESGQALERVGCRLQGVYSFEEKLNRHATVLPMRHNVPSLDK

TSWVAPSGMVSGSVTLGENSSVWYGAIVRGDFQPVVVGSNSNIQDAAYVGATSEFSGPVT

IGDNVSVGHGAVLKGCTVGDNVLIGMNSIISEHAEIQSGAVIAAGSYVEEGTTVPSGEVW

AGSPAKKLRDVRAGEAEYLKSLPGRYTELAGEHKGIMKVLKMKQAEYFA

>Cre3_Chlamydomonas_reinhardtii

MLKRVGQSLVPFARAGLTQTAESFRGVSSQFFDAPNGPSVKQVLIEDEWYNRQRSIFPLL

DKEPYYPVDVFVAPNAVVCGDVDIYGGASVFFGAVLRGDLNKIRLGNRSAILDRAVVHAA

RAVPTGLNAATLIGEKVTVEPYAVLRSCRVEPKVIIGARSVVCEGAVVESESILAPNSVV

PPARRIPSGELWGGSPAKFIRKLTDHERDRVLDDVSTHYHNLATMFRREALEPGTGWRDV

EAWRQKLVDQGEFQWINSREQKYLMPPAARGPRRLEKLTH

>Cme_Cyanidioschyzon_merolae

MSVFRRFLYHLGYLARETGQALDRAGCFLQGNFAYREALYLSRHRQIMNLVDRKPIISPQ

VQFIAPNAAIIGDVAIGAASSVWYGAVIRGDVNKVVIGERTNVQDRAVIHVASGGGKLER

ALPTFIGNEVTIGHGAILHACAVEDQAVVGMGAIVLDGSRVESGAVIGAGSVLPPGTVVG

AGQLWLGTPARFVRLVSAEEKQQFAVQCSQYVELAKMHATECGKTPDQLDAEQMAALLWE

ERSEDYLSSLGLLGKEEDVMAAQKAYLAHERQLAASGAPKGSGKPETKISSSTDARQQVS

AGH

>Cpa1_Cyanophora_paradoxa

MARKLGLLIRETGQALDRLGMRIMGDYGFRENLSRHRVLMNIYDKKPSVADGTFIAPSAS

VIGDVKIGPKSSIWYGSVVRGDVNYVTIGEETNVQDRCVIHVAKIAGNNPTKIGNKVTIG

HGAVIHACTIEDEVIIGMGATVLDGAVVQKHAIVAAGAVVPPGKTVPSGELWAGNPAKFL

RTVTDAEKAFFTKSATEYTKLAEDHADEWKKTHGAARGG

>Cpa2_Cyanophora_paradoxa

LGRTAARHAAGQRLGGVRASWTDAISKHRKVVVFNEKQPIVATDTYIAPSALVLGQVEVG

VRSSIWYNAVVRGDLNEVRIGGVSNIGDCAVVQTAPDNAENLMGGSTIIGNYVSIGAGAT

LRACVIENSVIIGARSVVSDGAVVERNSILEPGSVVVEGQRIPEGELWGGNPARFVRKLS

ESERADIDVVADKVYQINQDHGEEFYLPYSTAYLEL

>AcCa1_Acanthamoeba_castellanii

MMRGLLRRLRPSTPAAVLPTRNGADIPDVPLEKFGLTVPVQATAYNDLYNKHTTLVNLPG

KRPQISSESFVAPSATLVGNVEVWDRASVWYDCVINADTKLIRIGAGTNVQDGTVITEAD

EELTEDHDGSTIVGHWVTIGHRCVLKACTIEDHCLVGMGSVLGAGSYMESHSILGAGSVL

PAWQRIPSGQIWVGNPAKYLRDLTEEEFDFLEKSSAHYTVLSKQHAYEFYLPGHAYIDAE

KKGIQVGYQVEPLSGEESVLLAPNYKEKVSVH

>AcCa2_Acanthamoeba_castellanii

MLKRFSYVLGNTVRETAYALDRVGCRLQGNYAFTEELSRHRRVMGLYDKQPAISQDVFIA

PNASVIGSVSLGEGANVWYGSVLRGDVNDISVGKKSSIGNRSVVHASGGLTTLAPTKIGD

NVVVGDGVVLHGCTLEDECRVDDGAVLNDNVVVEKHAIVGPGAVVTSGKRVPSGQVWAGN

PAKYVRDVSEEEKEFAGWAEKRYTQAKAHLAQTIKLAEEKEVDLLTEDILREMRPGTRFA

D

>Ddi1_Dictyostelium_discoideum

MIKQLTKLNITKQLANNIGKRQYCSYFENRNNNLPYNTDPIEKVEDSPNTSSKYFDLYNK

HKTFVPFFDKNSITTPAITGLYPREGGEFVAPSASIIGNVNLGVGSSVWDNCVIRADVNY

IHIGAFTNVQDGTIIREANEPISLDHNGSTIIGDQVTIGHSCILEACTVEENCLIGMGSI

LEPESYVEANSILGSNSILTKGSRIKSGELWVGKPAKFVRNLTENEKIDISNSAHSYMLN

AEKAFESFGLDKDSFIYIDAQQQGIQVGWKGSYFSE

>Ddi2_Dictyostelium_discoideum

MSQKGLFGILGEVVKNTGLILHRTGCKMQGDYAYVEKLNRHTRLTAFGDNAPIVGQKSFI

APNASIIGDVVIGKESSIWYNAVLRGDVNSIHIGDKTVVSDRTVVHCSSNGPLGPKPTQI

GDKVYIGPGSIVHAATILGESFIGTGSTLCDGSVVEKNGFLEAGSLLTAGKTIKSGEYWG

GSPAKFIRQVTKDDESQLEKIIEQNINLSEQHEKQTSKSAKELNNDLLQKYVKNRTRSDH

ILNNPL

>Hve_Hartmannella_vermiformis

EAKSKYGKYAYLFEEKIPGTVLFADKWDKHKTIVRLDKKLNPKLSPETFVAPSATLAGNV

EVWDKASIWYNVTIRGDVKLVRIGAWTNVQDNTVITEAFQPIGADHDGSTIIGHYVTIGH

GCQLRACTVEDGCLVGMGSILSEGSYMEKNSMLGANSVLLSHDRVSTGEFWAGNP

>Had_Hyperamoeba_dachnaya

NYAFKEKLCRHSRLMPIYGKHPFIEEGTYVAPNASVIGDVDVGEKSAVWYGAVLRGDINS

IKIGDFTSIGDRVVVHVARENPKGPLPTVVGDHCVVEQGSILHACTLEDESFVGTGSILY

DGSRLSKHAILEPGSVLTAGKVVPSSQVWGGSPARFIRNATPDEVANIRSAAEFYSSLAQ

KHSAEVSKTEGEREMERVRDEVSPSNPYPEENLESIPQNTKSSSPKH

>Ehi_Entamoeba_histolytica

MSSQQFIPHNGNVPKVAKDAFITPGVFLIGDVEVESKASIWFNAVLRGDMAKIVIGENSN

VQDCSVVHTSIGKPTIVGKNVTIGHSVILHSCEVGDGSMIGMGSTILDDVKIGKNVLIGA

NSLVTSRTVIPDNSLVMGSPAKVVRELREKEFEYLKENIKEYDDIKQGYHLEQPQNQ

>Ppa1_Polysphondylium_pallidum

MTYCVNNVAKCLEILMVSRSSTVSNRRNFCTYYNSKDNQQHYDISELKKYSENENQVEKY

LDKYNKHQTLMPFYNKHSIALPTVFGLYPRTPLGTFVAPSASVIGNVVLCYGSSVWYNSV

IKADVNLIHIGNFTNIQDGTVIREAARPLSLDHDGSTIIGHYCTIGHNCVLEACTVEENC

LIGMGSVLEAGSYVETNSILGANSLLPKGARVPTGELWAGRPAKFVRKLTDEEMIDIHNQ

AAQYHKYSQSHHQDLGLHNPSSAYVDAENQGIEVGFKGEYF

>Ppa2_Polysphondylium_pallidum

MQGNYAYVEKLNRHSRIMPLRGKIATLGRNSFVAPNSSVIGDVQIGNQSSVWYNTVLRGDV

NSIVIGDESVISDRSVVHCSSGNGPKGAQPTVVGNRVYVGPGSIIHACKIEDDVHIGAGSI

VYDGAVVEAGAQLEAGSLVTAGKRVPAGQLWAGSPAKFVREVSAADKEMHQLTISDNNTLS

AEHEVQTSKSAKQQHIDQLENFAHRRERPENILNPATAHN

>Tth1_Tetrahymena_thermophila

MKLFRALTKSGLIQKIRQATGSEISTATKYGENISKHRSLMSLYDLHPQIGYQSYIAPNS

TVIGEVTIGNETTVWYNSVIRGDVNAVQIGNNVSIGENVVIHTAGSLPTGQPASVDIGHY

VIIGSKSTIYSCTIQDEVVIGQGCVILEGARIEKGAMIAANSVVPPGRLIPAGTLWAGNP

CTFVRNLTKSELATNIDHAKKQLHLAQQHRYEYLPYNSAYLQKSNSEEDLNPTKYDDVTI

NYNFGDEERAQENPLKY

>Tth2_Tetrahymena_thermophila

MKLFQAMWTRTIYSIGRMVRETGLALDRYGCKLEQDISCYEPLSRHRNILPIYDLVPTFY

HSTFIAPNSSLIGAVYLGQNTVVGYGSTLRGDNHAIRVGHNTVIGDKVAISNVATLAAGI

PVSTNIGNHVNIGAGCVLQSCVVDDNVTVGHNTVILEGSVLERGSVIAPNSLVPAGRLIP

SGQLWAGSPVRYVRDLKEEEIKLNLEQTEQNLSIGKTHKSSLIQQEAYDRLLA

>Tth3_Tetrahymena_thermophila

MLRLRLFDAYEKISMTFLGPLYRRIGKSLAQTGLNIQQPYTSDDRLVPSLRNIRVTNKIP

SINDSEFIAPNSVVIGDVITKEGSSIWYGATLRGELGPIEIGKQTVIQDLVNIQSGKQNQ

KTQIGDNVFIGPNSYIQSSKINDNSFVGMGSTVSTGCNLASNAVVAAGSVVPENTQVPSN

QIWAGSPAQYLRDITPEERQVLQEHHQECVQLARIHAEETEKSFREVLNDFDRITAEAEY

DHESLALQKMRDLGFPMEGEEEEYIEQRVFMREQLPPLESEFWKKNYDPYEQDLFHFPDS

FKAYQQQYKRYDEAKKYFEENPNVEATIIDREFKEPTNKKPWTRKY

>Ehu_Emiliania_huxleyi

MKRVLVGVGKALRDTGQAVERMGMRAQDNWIFQEKICRHRALMNLFDQRPKLRPSVFVAP

NASLIGNVSVMDESSIWYGAVVRGDQSPVDIGGKSSIGDRSVVLSASVNPTGFAAKTSIG

DWVTVGQGCVLRGCTVDNFAVVGDGCVIGEGALVETHGVLEAGSVLPAGGLVPRGEVHGG

NPAAFVRKLEKDEIAAIEKKAEDVSMSAKKHADEFLAYSNTYQLREQLGTAAGKI

>Plu_Pavlova_lutheri

MLRETGQALDRLGLRSQNNFVFRDKLSRHRAVMNLYEKRPSMAADVFVAPSASVIGDVIL

NDGVSVWYGAVVRSDVNPVTIGGYTNIQERAVVHAATSTPTGFKANCSIGSWVSVGQGAV

LRACTVEDYCVIGAGSVLLEGSLVEKHAIVEPGSVLPAGGRVPSGEMWGGNPIAFVRKLS

KEEAADIEKQANAVAD

>Pin1_Phytophthora_infestans

MMRKVTFELGRCVRETGQALDRLGLRVLNDNSFKEKFSRHRQVMALYDKRPRIAHDVWVA

PNATVVGDVEICNDASVFYNVVIRGDLNQVRIGNRTNVQDRTVIHTASSTSPGLAPGANI

GNDVTIGHGCTLYSCTVENNSLIGMGSIILDGALVESNTIIAAGSVVPPGRRIPSGQLWA

GNPAKYVRDLSDDEVADIAKQASEYKSIASTHSDEFLPYGTAYLDAEKIKAAGGHL

>Pin2_Phytophthora_infestans

MWSKQAGKAIRSLGQTIDRVGVSLEGKLAYTEHLNPSTRAVKNLGRSPKFEEGVFVAPNA

AVIGDVKVGKGSSIWYNATVRGDVNHITIGENTNIQDQAVVHVAKIHKDIPTKIGNNVTV

GPAAIVHACTIQDHCIIGTGAQVLDGAVVGAKSIITAGSIVTKGKQVPSGQLWSGVPARY

LRDLTAEETQFMQQCSSEYAQLAEQYADECAKTFEEYEADTERYKILRDVGETGLPQKGD

EREDTGLYFRY

>Ptr1_Phaeodactylum_tricornutum

MSSLVARASNGLAGMMRKVGSAFDSMGKGLEITKYTEKLVPSTRFVAVDGMVPKISDKGA

FVAPSAAVIGDVTIGKASSIWYGATVRGDVNTITIGDYTNIGDRAVVHVARIQGDFATSI

GNNVTIGAGALIHAATLKDNCVVGESAQVLDGATVESNVIIAPAAIVTPGTMIPSGELWA

GSPAKMIRVLTEDEIAAIPKQASETAALASMHAIEHSKSYEQVMEEEQVAENELYREVPV

PKQTENPLGDVLGQGMPGRIFRSTLSHPEDIYKGQQPK

>Ptr2_Phaeodactylum_tricornutum

MAKQSVSKAVTAYLGRALRETGAALKHRGEMEIFSRHRPKMTFLGKVPFVTNDTFIAPSA

SVIGDVTNWDQSSVWYKAVVRADSEHSITIGFCSSVGEGTVVNTLSSTGQLETGLPPDTY

IGHYVTVGAGCVLKSCRVDDLVVVGDKCTILEGSLVENHVILKPGTVVMPYQRIPSGQMW

AGNPAAFVSELTPDEKEDIQQQALKIFTSTKEHILEFLPYGRTFVHLEELEKQAGLQVKQ

G

>Gth1_Guillardia_theta

MAWAAGQALRETGVALERLACRMLGDLTYKEPLSCHRNVMRIFSDAPKIKEGCFVAPTAS

VIGKVTLGTNSNVWYSAVVRGDRSNISIGNNCNVMERAVLNPTSGEIAIGDNVTVGAGAV

IRAAKIGSGCMVGASAVLEDSVVVEDGAAVGPGAVVPASTVVPAGQIFTSAGLRALKADE

LAAIAAICGNVSKMAPVHTAECNKSFKDIEKEKEGYEWQQPLEDGDPMGLLSQDHEKSLQ

WQSKFT

>Gth2_Guillardia_theta

SALRSLGATLEKAGQALAGSSREVLSRHREVMALKSHAPSIHRTCFVAPSANVVGNVKLA

EKSSIWYGAIVRGDLASISIGSMSSIADKATISPMGEGSVQIGNRVLVGQGAVVGVATIH

DDAVIGMGSTIGDRAVIESGAYIAPGSVVASGTVVPKAKLFCGEQVLRDLTPAESARLAS

SVESLCFLRLEHAAEVYKDVIDIEQDKSDAKWMEERSLDYDSSLGLLRTMRGHRFGKHCG

RGRPDG

>Gth3_Guillardia_theta

MLRKLNVGGCFRQMGGIARLQHDRFPPLRRMAVYSLGEHHPTLEKETWVAPNAAVIGKVK

MEQGSSVWFSATLRGDNELIHVGKDSNIQDGCVVHTDIGFPVNIGQRVTVGHKVMLHGCQ

IGDDTLVGIGSTILNGAKIGKGCLIGAHSLVLENTEIPDGSLVLGSPAKVVKEVSAVMRE

AMKSGPLTYKHKAMEFEKDLKEVKD

>Gth4_Guillardia_theta

MLRGNMLKKLMPVGVKIVXPFPVRHLQSMNNPGNSFLDPKEAEKPEDRYSEAGKLMNMDV

KTgIVELSKAFHRHRVSIPIGTAIPDIAQGTFVAPDATIVGDVTLGDNSAVYYGSVIRGD

EGPVLIGFRCQVGENSVITSDSDMTDISIDTDESGGRLEDLEKSVTIGHYVTIEPGCYLR

SCTIQDRVVIGANSVICEGALVEAGAQVGPGSIVPPGRRIPANEVWQGRPAQYVRTLTGS

DSEDLDKKLKTFVKDTELHIDCNYgIGEEHNMLHRDAMEAMQKGN

>Bho_Blastocystis_hominis

CGKCVERCACQAQDAAKFTETYSRHRPVLPINGKMPVIDPTVYIATNATVSGNVQIATGS

AVWYGSIVRGDNNSISIGTESHIQDRSVVSSVKSTDSGLPGSVSIGNNVVVGYGSVLTGC

RIDDNCHIGSCCRILEGAHMETNSSLASGSVVEQGKTIPAGEYWAGNPAKFVRKVGEHEK

DDMLQYSRDCTE

>Ram_Reclinomonas_americana

MLSRVLVGRGLRAFASAGASGESSNAAPVAGQVLKRLYEQYREPYSRHRSLIVLDDERRP

SVSVEAYVHPSATLVGNVFVSDRASVWPGCVLRGDVGRIMIGAYSNLQDGVVVTAPGETR

ADGSAPVTSIGDFVTIGHNAILHGCTLTKETLVGMGAIVLEGCVMEPQSMLAAGTVLLAG

TRVPAGQLWAGNPGKYVRDLTDDEIHFIAVSADVYYASAQQITAATAVNSLAYVE

>Sec_Seculamonas_ecuadoriensis

MLSRVFSSLRLARTDAPVYQRYTAAAGQRLKQLYEEFREPYSRHRTLISLSDTLPYVSHD

AFVAPSAVCAGAVVVADRASVWYGAVVRGDRAGVHIGAYTNVQDGCVITTGAGKPTMIGD

YVTIGHDALLHGVTLESESFVGMGAILMEGVVVEKHAMIAAGAVVPPGTRVPAGQLWAGN

PARYLRDLTYDEIDFITKSAEEYYALSKGARATFAPGSLNYVQVEKIRARIGEPPLMQPS

ENPCAN

>Mja1_Malawimonas_jakobiformis

MLRAVLNGLGKALRETGIAAERTAAALQHNPAVKENYSRHRQVMPIDDKRPEISVDSYVA

PNAAVIGDVFVNDKASVWYSSVVRGDMSYVNIGAYSNVQDRAVISTSETTDDADGGVSIG

NYVTVGHGAVLHACKIEDEATIGMGAILQQGVHVGKNAFVAAGSVVEAGTKIPEGQLWAG

NPAQFIRELTDREREQH

>Mja2_Malawimonas_jakobiformis

CTSSLQLLIMTEKVIRGIANAVAPPLRAFGRAMDSLGVALQGQRAYVETLDRSSRFVPLK

SRKQTPSVGQSVFVAPNASVVGDVKVGGGSAVWYGAVVRGDVNSIRIGNHSHVMDQAVIH

VSSGKNMKGAAQPTVLGNNVIVGSGATLHGCTVQDNAVIGAGAIVLDGAVIEEGAVVAAG

AFVPANTVVKAGTVFGGSPAKEKGVVTQEDVAAYRERHAALQKAAQKHTAEHAKTGSQYA

QE

>Ngr1_Naegleria_gruberi

MKRGTNASTAQARAVRSTGLDDQDVLSGKQHILHKQRKDRLVFLPDREILENSNYPAEYP

STYVPVPEPEVPNHKGRRALGQLYKQFLELYNRHRRFVWFNGYDPSIQAGGVWVAPSATV

IGDVRLCDHVNVWYNAVLRGDKNSIEIGGYTNIQDGVVITTDDKPNFGGFDSNVVIGGHT

TIGHGVKLHACRIGNECVIGMNATILEGAVIEDNVVIAAGSLVPPGRRIPHGEMWAGSPA

KFVRKLGHHEEEQVKTDAEAYVNLAEAHSLEFTSFGKAYKEVDVIADKLEQINPESVDGR

PVHWQVWHEQSKNATVALWKKDRIL

>Ngr2_Naegleria_gruberi

RMNVPKQSMRHLIGSLFREAGEAMDRVGCFLQGSLAYKEDLNRTRRVMKFKNFKPSVQPS

SFIAPNASVIGSVSLGPNSSVWYNVVIRGDVNSIQIGENTNIQDRVIIHCTGKVGHEKPT

IIGNNVTVESGAILHACTLEDESYIGFGATVLDGAVVGRGAMIAPGAVVTPGTIVPGGEI

WAGVPAKKLRELTPEEQESIKKSAAELSELAQVHKQEQDKEFEELLHDMETFKFREDRLE

EYTYEKVESPSSTAPTKN

>Egr_Euglena_gracilis

MVGIHWDRSAGGRWTPNDKFPLFDYEFPIHPGRIILRWLYKQGKEPVNMQRSILVTDDFA

TPSVYPFGWHAPSAILIGDACISNDAAVFDHCVLRADRAAIWVGPKSHVLEGCTLTTAPP

TPDRPALGSVLIGENTVVGAGSSLNACWIGDHCIIGSGCTIGFGARIDDGAVVGAGSVVE

DDQYIPAGEVWVGRPARYLRKTGDVDTFTAVAENDTLRSLHLAYSEYETTHGNVWAESDK

VC

>Tbr1_Trypanosoma_brucei

MKRCRLALAEAQMPAVLPDWAVKKPTKLALALDRLATKLSVVSRKIISLSDALMGVQPRE

ALNCVPRLLAVNGVRPTVMDNVFIAPSAFLSGDVRVGRKNYIGYNAIVRAERGETIYFGE

SCNVQEKAIVTGGTTIGKWTTIEPMAIVDAADIASCSFVGANAIVMRDAKIESGSMLCAA

SVLQSGAVIPSGEMWAGNPAEKVRDLTEKEQDDMIKAAKHMVLLAIEHRDSWELTWEELE

DQREAREQFARYAENNREVRTKPMYIKEPPRPSRKAMSRKTPQEMVDGGEHKPPLAESIQ

QGY

>Tbr2_Trypanosoma_brucei

MPATIVSGVAPYFAFMLKDIGARLAIMSHYLKTKRHMYPVTRHQNVRAYRGMLPWTQDSA

FIAPTAFVSGNVSLGHDTCIFYHTVIRNYNIRDETAIGDHTVVMDRVSFLGQVRVGGGVY

IGPGSTLDCCTVGDNAYIGAGASIALGAVVENNAIIAAGSHVPKDTHVYAYELWAGNPAQ

KVEEVSPDQVAEVASIVHDQIAVGKAHAHAIHEHMHHTAELDAEWLHHALEAMEKQQQQM

ALKLPVDIPLEAKRFLTPRVHMRRPEMHMRMSYPVNRIAPWMPKVADQTANA

>Bna_Bigelowiella_natans (EST with unexcised intron???)

MISRLPAAASTLANIGRCIFAPSQIIIRRGRGWRQQCKASGMATTADAAAGRGIIEMQAP

QIDNDAYVAKGAILVQNVKVSEGASIWYNCVLRGDVASIQIGKNTNIQDGTVIHVASEQQ

GTKGKLNTIIGDGVTIGHMALLHACIIEDNAFVGMKACVMDGAVVKTHGMLAAGALLTPG

KIVGTGELWAGQPAKLMRKLTEKEIENIYKSAQTYAKLAACYRNNLLTEFSSN

>CAM_Methanosarcina_thermophila

QEITVDEFSNIRENPVTPWNPEPSAPVIDPTAYIDPQASVIGEVTIGANVMVSPMASIRS

DEGMPIFVGDRSNVQDGVVLHALETINEEGEPIEDNIVEVDGKEYAVYIGNNVSLAHQSQ

VHGPAAVGDDTFIGMQAFVFKSKVGNNCVLEPRSAAIGVTIPDGRYIPAGMVVTSQAEAD

KLPEVTDDYAYSHTNEAVVYVNVHLAEGYKETS

>AL_gi|88607111|ref|YP_505730_1| AL_gi|88607111|ref|YP_505730.1| hexapeptide transferase family protein [Anaplasma phagocytophilum HZ]

MREVLVPYAGVSPSVDSTAFIAGNARIIGDVCIGKNASIWYGTVLRGDVDKIEVGEGTNI

QDNTVVHTDSMHGDTVIGKFVTIGHSCILHACTLGNNAFVGMGSIVMDRAVMEEGSMLAA

GSLLTRGKIVKSGELWAGRPAKFLRMMTEEEILYLQKSAENYIALSRGYL

>AL_gi|73667471|ref|YP_303487_1| AL_gi|73667471|ref|YP_303487.1| hexapaptide repeat-containing transferase [Ehrlichia canis str. Jake]

MHNIISYGIFVPNIDGTVFVASTASIVGSVYISKNASIWYNSVLRGDVGMISIGEGTNIQ

DNTVIHVDRNQGDTEVGKMVTIGHGCILHACQIHDYVFVGMGSIIMDKVIMEENTMLAAG

SLVTKGKVIKSGELWAGRPAKFFRMLSEEELNHIKESADNYIRLSQEYLECR

>AL_gi|83593755|ref|YP_427507_1| AL_gi|83593755|ref|YP_427507.1| hexapaptide repeat-containing transferase [Rhodospirillum rubrum ATCC 11170]

MSASEPPAVPLGTPAAAGPIILPHRGIWPRIASDAFIAPGAVVIGDVEIGARTSVWFGCV

LRGDVHHIRIGARTNIQDGTIVHVTGGHLGTLIGDDITIGHRALLHACTLESNCFVGMGA

IVMDGAVVESWAMVAAGALVTPGKRVESRSLWAGSPAARKRDLSAEDIAFFPESARKYAD

LADIYVEEM

>DE_gi|197121924|ref|YP_002133875_1| DE_gi|197121924|ref|YP_002133875.1| transferase hexapeptide repeat protein [Anaeromyxobacter sp. K]

MPILLPYAGARPRLHPSVFAAPGCVVTGDVEVGPEASLWFGTVVRGDVNTVRIGARTNVQ

DGTVIHVTTRTHPTVIGEDVTIGHRAVLHGCTVHDRCLIGIGAIVLDGAVVGPDAMVGAG

ALVPPGAVVPPGTLVMGQPAKPKRPLTPEEIAFLRTSAANYVSYAARYRAEGGVP

>AL_gi|83310891|ref|YP_421155_1| AL_gi|83310891|ref|YP_421155.1| carbonic anhydrase/acetyltransferase [Magnetospirillum magneticum AMB-1]

MSGTILPFEGTSPTIAPDVFVAPTAVVIGDTVIGAGTSVWFNCVIRGDVHEIRIGERTNI

QDGTVIHVTGGKLGTYIGSDITIGHGAILHACTLEDACFVGMGAVVLDGVVVESGAMVAA

GAVVTPGKRVKAGELWGGNPAKLLRRLSDEEIAFFPVSAEKYVELAAKYFKA

>AL_gi|57239548|ref|YP_180684_1| AL_gi|57239548|ref|YP_180684.1| hypothetical protein Erum8210 [Ehrlichia ruminantium str. Welgevonden]

MNIFNYMQIMPNISVDAFVAPTAVIIGDVCVSDKCSIWYNSVLRGDVGQIVIGVGTNIQD

GTIIHVDRKYGNTNIGKKVTIGHGCILHACEIQDYVLVGMGSIIMDNVVVEKNAMVAAGS

LIVRGKVVKTGELWAGRPAQFLRMLSSDEIEEISKSADNYIELASDYITGKL
