# Supplementary material for: Evidence for an early evolutionary emergence of γ-type carbonic anhydrases as components of mitochondrial respiratory complex I
Source: BMC Evol Biol. 2010 Jun 14;10:176. doi: 10.1186/1471-2148-10-176 (PMC2900272; doi:10.1186/1471-2148-10-176)
Supplement: Additional File 4 — Phylogenetically broad alignment of eukaryotic and prokaryotic γCAs.: A multiple alignment of eukaryotic and prokaryotic γCAs including complete and partial sequences from diverse eukaryotic groups. [file 1471-2148-10-176-S4.DOC]

**Additional File 4: Phylogenetically broad alignment of eukaryotic and prokaryotic γCAs**

CLUSTAL W (1.83) multiple sequence alignment

AcCa1 ------------------------------------------MMRGLLRRLRPSTPAAVL

AcCa2 ----------------------------------------------------MLKRFSYV

CA1 --------------------------------------------------MGTLGRAFYS

CA2 --------------------------------------------------MGTLGRAIYT

CA3 --------------------------------------------------MGTMGKAFYS

CAL1 -----------------------------------MATSIARLSRR----GVTSNLIRRC

CAL2 -----------------------------------MATSLARISKRSITSAVSSNLIRRY

Cre1 -MSLFKSSLPAGFLFPYRHPKAKGLVEGTLYGLGSLFRGVGAALDELGSMVQGPQGSVKD

Cre2 -----------------------------------------------MNPINGLKTILHR

Cre3 -----------------------------------MLKRVGQSLVP----FARAGLTQTA

Ddi1 ---------------------------------MIKQLTKLNITKQLANNIGKRQYCSYF

Ddi2 ---------------------------------------------------MSQKGLFGI

Iga ------------------------------------------------------------

Hve -----------------------------------------------EAKSKYGKYAYLF

Cpa1 -----------------------------------------------------MAR---K

Cpa2 ---------------------------------------LGRTAAR----------HAAG

Ehi ------------------------------------------------------------

Cme --------------------------------------------------MSVFRRFLYH

Gth1 --------------------------------------------------------MAWA

Gth3 ------------------------------------------------------------

Gth4 --------------------MLRGNMLKKLMPVGVKIVXPFPVRHLQSMNNPGNSFLDPK

Gth2 ------------------------------------------------------------

Sec ----------------------------------------------MLSRVFS--SLRLA

Ram ----------------------------------------------MLSRVLVGRGLRAF

Egr ---------------------------------------------MVGIHWDRSAGGRWT

Tbr -----------------------------MKRCRLALAEAQMPAVLPDWAVKKPTKLALA

Mja1 ----------------------------------------------------MLRAVLNG

Mja2 ---------------------------------------CTSSLQLLIMTEKVIRGIANA

Tth1 ------------------------------------------MKLFRALTKSG---LIQK

Tth2 ----------------------------------------------MKLFQAMWTRTIYS

Tth3 -------------------------------------------MLRLRLFDAYEKISMTF

Oma ------------------------------------------------------------

Ehu ---------------------------------------------M----------KRVL

Plu ------------------------------------------------------------

Pin1 ----------------------------------------------------MMRKVTFE

Pin2 -------------------------------------------------MWSKQAGKA--

Ptr1 -------------------------------------------------MSSLVARASNG

Ptr2 ---------------------------------------MAKQSVS----------KAVT

Ngr1 MKRGTNASTAQARAVRSTGLDDQDVLSGKQHILHKQRKDRLVFLPDREILENSNYPAEYP

Ngr2 ------------------------------------------------RMNVPKQSMRHL

Hda ------------------------------------------------------------

Bho ---------------------------------------------------------CGK

ARCH_Msa ------------------------------------------------------------

AL_gi|88607111| ------------------------------------------------------------

AL_gi|73667471| ------------------------------------------------------------

AL_gi|83593755| ------------------------------------------------------------

DE_gi|197121924 ------------------------------------------------------------

AL_gi|83310891| ------------------------------------------------------------

AL_gi|57239548| ------------------------------------------------------------

AcCa1 PTRNGADIPDVPLEKFGLTVPVQATAYNDLYNKHTTLVNLPGKR-PQISSES--------

AcCa2 LGNTVRETAYALDRVGCRLQGNYAFTEE--LSRHRRVMGLYDK--QPAISQDV-------

CA1 VGFWIRETGQALDRLGCRLQGKNYFREQ--LSRHRTLMNVFDK--APIVDKEA-------

CA2 VGNWIRGTGQALDRVGSLLQGSHRIEEH--LSRHRTLMNVFDK--SPLVDKDV-------

CA3 VGFWIRETGQALDRLGCRLQGKNHFREQ--LSRHRTLMNVFDK--TPNVDKGA-------

CAL1 FAAEAALARKTELPKPQFTVSPSTDRVKWDYRGQRQIIPLGQW--LPKVAVDA-------

CAL2 FAAEAVAVATTETPKPKSQVTPSPDRVKWDYRGQRQIIPLGQW--LPKVAVDA-------

Cre1 HVQPNLAFAPVHRKPDVPVNAGQVVPAP--PAAARTLKIKEVV-VPNK-HSTA-------

Cre2 VGFAMRESGQALERVGCRLQGVYSFEEK--LNRHATVLPMRHN--VPSLDKTS-------

Cre3 ESFRGVSSQFFDAPNGP-SVKQVLIEDEWYNR-QRSIFPLLDK--EPYYPVDV-------

Ddi1 ENRNNNLPYNTDPIEKVEDSPNTSSKYFDLYNKHKTFVPFFDKNSITTPAITGLYPREGG

Ddi2 LGEVVKNTGLILHRTGCKMQGDYAYVEK--LNRHTRLTAFGDN--APIVGQKS-------

Iga ------------------------------------------------------------

Hve EEK----IPGT-------------VLFADKWDKHKTIVRLDKKLNPKLSPET--------

Cpa1 LGLLIRETGQALDRLGMRIMGDYGFREN--LSRHRVLMNIYDK--KPSVADGT-------

Cpa2 QRLGGVRASWTDAISKH-----------------RKVVVFNEK--QPIVATDT-------

Ehi -------------------------MSSQQFIPHN--------GNVPKVAKDA-------

Cme LGYLARETGQALDRAGCFLQGNFAYREALYLSRHRQIMNLVDR-KPIISPQVQ-------

Gth1 AGQALRETGVALERLACRMLGDLTYKEP--LSCHRNVMRIFSD--APKIKEGC-------

Gth3 ------MLRKLNVGGCFRQMGGIARLQHDRFPPLRRMAVYSLGEHHPTLEKET-------

Gth4 EAEKPEDRYSEAGKLMNMDVKTGIVELSKAFHRHRVSIPIGTA--IPDIAQGT-------

Gth2 --SALRSLGATLEKAGQALAG--SSREV--LSRHREVMALKSH--APSIHRTC-------

Sec RTDAPVYQRYTAA--AGQRLKQLYEEFREPYSRHRTLISLSDT-LPYVSHDA--------

Ram ASAGASGESSNAAPVAGQVLKRLYEQYREPYSRHRSLIVLDDERRPSVSVEA--------

Egr PNDKFPLFDYEFPIHPGRIILRWLYKQGKEPVNMQRSILVTDDFATPSVYPFG-------

Tbr LDRLATKLSVVSRKIISLSDALMGVQPREALNCVPRLLAVNGVR-PTVMDNV--------

Mja1 LGKALRETGIAAERTAAALQHNPAVKEN--YSRHRQVMPIDDK-RPEISVDS--------

Mja2 VAPPLRAFGRAMDSLGVALQGQRAYVET--LDRSSRFVPLKSRKQTPSVGQSV-------

Tth1 IRQATGSEISTATKYG------------ENISKHRSLMSLYDL--HPQIGYQS-------

Tth2 IGRMVRETGLALDRYGCKLEQDISCYEP--LSRHRNILPIYDL--VPTFYHST-------

Tth3 LGPLYRRIGKSLAQTGLNIQQPYTSDDR--LVPSLRNIRVTNK--IPSINDSE-------

Oma ----------TRQRWSPVLLRRVAVSVG---SVRTATIRLGAT-CPSI-AESA-------

Ehu VGVGKALRDTGQAVERMGMRAQDNWIFQEKICRHRALMNLFDQ--RPKLRPSV-------

Plu -----MLRETGQALDRLGLRSQNNFVFRDKLSRHRAVMNLYEK--RPSMAADV-------

Pin1 LGRCVRETGQALDRLGLRVLNDNSFKEK--FSRHRQVMALYDK-RPRIAHDV--------

Pin2 ----IRSLGQTIDRVGVSLEGKLAYTEH--LNPSTRAVKNLGR-SPKF-EEGV-------

Ptr1 LAGMMRKVGSAFDSMGKGLE-ITKYTEK--LVPSTRFVAVDGM-VPKISDKGA-------

Ptr2 AYLGRALRETGAALKHRG--------EMEIFSRHRPKMTFLGK--VPFVTNDT-------

Ngr1 STYVPVPEPEVPNHKGRRALGQLYKQFLELYNRHRRFVWFNGY--DPSIQAGG-------

Ngr2 IGSLFREAGEAMDRVGCFLQGSLAYKED--LNRTRRVMKFKNF-KPSV-QPSS-------

Hda ---------------------NYAFKEK--LCRHSRLMPIYGK--HPFIEEGT-------

Bho CVERCACQAQDAAKFT------------ETYSRHRPVLPINGK--MPVIDPTV-------

ARCH_Msa ---------------------QEITVDEFSNIRENPVTPWNPEPSAPVIDPTA-------

AL_gi|88607111| --------------------------------MREVLVPYAGV-SPSV-DSTA-------

AL_gi|73667471| ---------------------------------MHNIISYGIF-VPNI-DGTV-------

AL_gi|83593755| --------------MSASEPPAVPLGTP--AAAGPIILPHRGI-WPRI-ASDA-------

DE_gi|197121924 -MPILLPYAGAR----------------------------------PRLHPSV-------

AL_gi|83310891| --------------MSGT------------------ILPFEGT-SPTI-APDV-------

AL_gi|57239548| ----------------------------------MNIFNYMQI-MPNI-SVDA-------

AcCa1 -FVAPSATLVGNVEVWDRASVWYDCVINADT-KLIRIGAGTNVQDGTVITEADEELT---

AcCa2 -FIAPNASVIGSVSLGEGANVWYGSVLRGD-VNDISVGKKSSIGNRSVVHASG-GLT---

CA1 -FVAPSASVIGDVHIGRGSSIWYGCVLRGD-VNTVSVGSGTNIQDNSLVHVAKSNLS---

CA2 -FVAPSASVIGDVQIGKGSSIWYGCVLRGD-VNNISVGSGTNIQDNTLVHVAKTNIS---

CA3 -FVAPNASLSGDVHVGRGSSIWYGCVLRGD-ANSISVGAGTNIQDNALVHVAKTNLS---

CAL1 -YVAPNVVLAGQVTVWDGSSVWNGAVLRGD-LNKITVGFCSNVQERCVVHAAWSS-----

CAL2 -YVAPNVVLAGQVTVWDGSSVWNGAVLRGD-LNKITVGFCSNVQERCVVHAAWSS-----

Cre1 -FVAANANVLGNVKLGAGSSVWYGAVLRGD-VNGIEVGANSNIQDNAIVHVSKYSMD---

Cre2 -WVAPSGMVSGSVTLGENSSVWYGAIVRGD-FQPVVVGSNSNIQDAAYVGATSEFS----

Cre3 -FVAPNAVVCGDVDIYGGASVFFGAVLRGD-LNKIRLGNRSAILDRAVVHAARAV-----

Ddi1 EFVAPSASIIGNVNLGVGSSVWDNCVIRADV-NYIHIGAFTNVQDGTIIREANEPIS---

Ddi2 -FIAPNASIIGDVVIGKESSIWYNAVLRGD-VNSIHIGDKTVVSDRTVVHCSSNGPL---

Iga --VAPNASLIGNVQIEDESSIWYGAVVRGD-QSPVSIGGRTSIGDRTVVASSSVN-----

Hve -FVAPSATLAGNVEVWDKASIWYNVTIRGDV-KLVRIGAWTNVQDNTVITEAFQPIG---

Cpa1 -FIAPSASVIGDVKIGPKSSIWYGSVVRGD-VNYVTIGEETNVQDRCVIHVAK--IA---

Cpa2 -YIAPSALVLGQVEVGVRSSIWYNAVVRGD-LNEVRIGGVSNIGDCAVVQTAPDN-----

Ehi -FITPGVFLIGDVEVESKASIWFNAVLRGD-MAKIVIGENSNVQDCSVVHTSIG------

Cme -FIAPNAAIIGDVAIGAASSVWYGAVIRGD-VNKVVIGERTNVQDRAVIHVASGGGK---

Gth1 -FVAPTASVIGKVTLGTNSNVWYSAVVRGD-RSNISIGNNCNVMERAVLNPTS-------

Gth3 -WVAPNAAVIGKVKMEQGSSVWFSATLRGD-NELIHVGKDSNIQDGCVVHTDIG------

Gth4 -FVAPDATIVGDVTLGDNSAVYYGSVIRGD-EGPVLIGFRCQVGENSVITSDSDMTDISI

Gth2 -FVAPSANVVGNVKLAEKSSIWYGAIVRGD-LASISIGSMSSIADKATISPMGE------

Sec -FVAPSAVCAGAVVVADRASVWYGAVVRGDR-AGVHIGAYTNVQDGCVITT---------

Ram -YVHPSATLVGNVFVSDRASVWPGCVLRGDV-GRIMIGAYSNLQDGVVVTAPGETRA---

Egr -WHAPSAILIGDACISNDAAVFDHCVLRAD-RAAIWVGPKSHVLEGCTLTTAPPTPD---

Tbr -FIAPSAFLSGDVRVGRKNYIGYNAIVRAERGETIYFGESCNVQEKAIVTG---------

Mja1 -YVAPNAAVIGDVFVNDKASVWYSSVVRGD-MSYVNIGAYSNVQDRAVISTSETTD----

Mja2 -FVAPNASVVGDVKVGGGSAVWYGAVVRGD-VNSIRIGNHSHVMDQAVIHVSSGKNM---

Tth1 -YIAPNSTVIGEVTIGNETTVWYNSVIRGD-VNAVQIGNNVSIGENVVIHTAG-----SL

Tth2 -FIAPNSSLIGAVYLGQNTVVGYGSTLRGD-NHAIRVGHNTVIGDKVAISNVATLAA---

Tth3 -FIAPNSVVIGDVITKEGSSIWYGATLRGE-LGPIEIGKQTVIQDLVNIQ-----SG---

Oma -FVAPSADLIGDVRIHEDVSVWYQCVLRGD-VAPIQVGARSNIQDGTIVHAASAELN---

Ehu -FVAPNASLIGNVSVMDESSIWYGAVVRGD-QSPVDIGGKSSIGDRSVVLSASVN-----

Plu -FVAPSASVIGDVILNDGVSVWYGAVVRSD-VNPVTIGGYTNIQERAVVHAATST-----

Pin1 -WVAPNATVVGDVEICNDASVFYNVVIRGD-LNQVRIGNRTNVQDRTVIHTASSTS----

Pin2 -FVAPNAAVIGDVKVGKGSSIWYNATVRGD-VNHITIGENTNIQDQAVVHVAKIHK----

Ptr1 -FVAPSAAVIGDVTIGKASSIWYGATVRGD-VNTITIGDYTNIGDRAVVHVARIQG----

Ptr2 -FIAPSASVIGDVTNWDQSSVWYKAVVRADSEHSITIGFCSSVGEGTVVNTLSSTG--QL

Ngr1 VWVAPSATVIGDVRLCDHVNVWYNAVLRGD-KNSIEIGGYTNIQDGVVITTDDKPNFG--

Ngr2 -FIAPNASVIGSVSLGPNSSVWYNVVIRGD-VNSIQIGENTNIQDRVIIHCTGKVG----

Hda -YVAPNASVIGDVDVGEKSAVWYGAVLRGD-INSIKIGDFTSIGDRVVVHVARENPK---

Bho -YIATNATVSGNVQIATGSAVWYGSIVRGD-NNSISIGTESHIQDRSVVSSVK-----ST

ARCH_Msa -YIDPQASVIGEVTIGANVMVSPMASIRSDEGMPIFVGDRSNVQDGVVLHALETINEEGE

AL_gi|88607111| -FIAGNARIIGDVCIGKNASIWYGTVLRGD-VDKIEVGEGTNIQDNTVVHTDSMHG----

AL_gi|73667471| -FVASTASIVGSVYISKNASIWYNSVLRGD-VGMISIGEGTNIQDNTVIHVDRNQG----

AL_gi|83593755| -FIAPGAVVIGDVEIGARTSVWFGCVLRGD-VHHIRIGARTNIQDGTIVHVTGGHL----

DE_gi|197121924 -FAAPGCVVTGDVEVGPEASLWFGTVVRGD-VNTVRIGARTNVQDGTVIHVTTR------

AL_gi|83310891| -FVAPTAVVIGDTVIGAGTSVWFNCVIRGD-VHEIRIGERTNIQDGTVIHVTGGKL----

AL_gi|57239548| -FVAPTAVIIGDVCVSDKCSIWYNSVLRGD-VGQIVIGVGTNIQDGTIIHVDRKYG----

AcCa1 ---------EDHDGSTIVGHWVTIGHRCVLKA-CTIEDHCLVGMGSVLGAGSY----MES

AcCa2 ---------TL--APTKIGDNVVVGDGVVLHG-CTLEDECRVDDGAVLNDNVV----VEK

CA1 ---------GKV-HPTIIGDNVTIGHSAVLHG-CTVEDETFIGMGATLLDGVV----VEK

CA2 ---------GKV-LPTLIGDNVTVGHSAVIHG-CTVEDDAFVGMGATLLDGVV----VEK

CA3 ---------GKV-LPTVIGDNVTIGHSAVLHG-CTVEDEAYIGTSATVLDGAH----VEK

CAL1 PTGLP--------AATIIDRYVTVGAYSLLRS-CTIEPECIIGQHSILMEGSL----VET

CAL2 PTGLP--------AQTLIDRYVTVGAYSLLRS-CTIEPECIIGQHSILMEGSL----VET

Cre1 ----------GTARPTVIGNNVTIGHAATVHA-CTIEDNCLVGMGATVLDGAT----VKS

Cre2 -------------GPVTIGDNVSVGHGAVLKG-CTVGDNVLIGMNSIISEHAE----IQS

Cre3 PTGLN--------AATLIGEKVTVEPYAVLRS-CRVEPKVIIGARSVVCEGAV----VES

Ddi1 ---------LDHNGSTIIGDQVTIGHSCILEA-CTVEENCLIGMGSILEPESY----VEA

Ddi2 ---------GP--KPTQIGDKVYIGPGSIVHA-ATILGESFIGTGSTLCDGSV----VEK

Iga PTGFA--------ARTAIGDWVTVGEGCVLRG-CSVDNYAVIGEGCIIQEGAL----VES

Hve ---------ADHDGSTIIGHYVTIGHGCQLRA-CTVEDGCLVGMGSILSEGSY----MEK

Cpa1 ---------GN--NPTKIGNKVTIGHGAVIHA-CTIEDEVIIGMGATVLDGAV----VQK

Cpa2 AENLMG-------GSTIIGNYVSIGAGATLRA-CVIENSVIIGARSVVSDGAV----VER

Ehi -------------KPTIVGKNVTIGHSVILHS-CEVGDGSMIGMGSTILDDVK----IGK

Cme ---------LERALPTFIGNEVTIGHGAILHA-CAVEDQAVVGMGAIVLDGSR----VES

Gth1 -------------GEIAIGDNVTVGAGAVIRA-AKIGSGCMVGASAVLEDSVV----VED

Gth3 -------------FPVNIGQRVTVGHKVMLHG-CQIGDDTLVGIGSTILNGAK----IGK

Gth4 DTDESGGRLEDLEKSVTIGHYVTIEPGCYLRS-CTIQDRVVIGANSVICEGAL----VEA

Gth2 -------------GSVQIGNRVLVGQGAVVGV-ATIHDDAVIGMGSTIGDRAV----IES

Sec ----------GAGKPTMIGDYVTIGHDALLHG-VTLESESFVGMGAILMEGVV----VEK

Ram ---------DGSAPVTSIGDFVTIGHNAILHG-CTLTKETLVGMGAIVLEGCV----MEP

Egr ---------RPALGSVLIGENTVVGAGSSLNA-CWIGDHCIIGSGCTIGFGAR----IDD

Tbr --------------GTTIGKWTTIEPMAIVDA-ADIASCSFVGANAIVMRDAK----IES

Mja1 ----------DADGGVSIGNYVTVGHGAVLHA-CKIEDEATIGMGAILQQGVH----VGK

Mja2 ---------KGAAQPTVLGNNVIVGSGATLHG-CTVQDNAVIGAGAIVLDGAV----IEE

Tth1 PTGQP--------ASVDIGHYVIIGSKSTIYS-CTIQDEVVIGQGCVILEGAR----IEK

Tth2 ---------GIP-VSTNIGNHVNIGAGCVLQS-CVVDDNVTVGHNTVILEGSV----LER

Tth3 ---------KQN-QKTQIGDNVFIGPNSYIQS-SKINDNSFVGMGSTVSTGCN----LAS

Oma ----------GTPRGTTIGEDVTVGHGAVLHA-CTLKDRCFVGMRAVVMDRAV----VET

Ehu PTGFA--------AKTSIGDWVTVGQGCVLRG-CTVDNFAVVGDGCVIGEGAL----VET

Plu PTGFK--------ANCSIGSWVSVGQGAVLRA-CTVEDYCVIGAGSVLLEGSL----VEK

Pin1 ---------PGLAPGANIGNDVTIGHGCTLYS-CTVENNSLIGMGSIILDGAL----VES

Pin2 ------------DIPTKIGNNVTVGPAAIVHA-CTIQDHCIIGTGAQVLDGAV----VGA

Ptr1 ------------DFATSIGNNVTIGAGALIHA-ATLKDNCVVGESAQVLDGAT----VES

Ptr2 ETGLP--------PDTYIGHYVTVGAGCVLKS-CRVDDLVVVGDKCTILEGSL----VEN

Ngr1 ----------GFDSNVVIGGHTTIGHGVKLHA-CRIGNECVIGMNATILEGAV----IED

Ngr2 -----------HEKPTIIGNNVTVESGAILHA-CTLEDESYIGFGATVLDGAV----VGR

Hda ---------GP--LPTVVGDHCVVEQGSILHA-CTLEDESFVGTGSILYDGSR----LSK

Bho DSGLP--------GSVSIGNNVVVGYGSVLTG-CRIDDNCHIGSCCRILEGAH----MET

ARCH_Msa PIEDNIVEVDGKEYAVYIGNNVSLAHQSQVHGPAAVGDDTFIGMQAFVFKSKVGNNCVLE

AL_gi|88607111| --------------DTVIGKFVTIGHSCILHA-CTLGNNAFVGMGSIVMDRAV----MEE

AL_gi|73667471| --------------DTEVGKMVTIGHGCILHA-CQIHDYVFVGMGSIIMDKVI----MEE

AL_gi|83593755| --------------GTLIGDDITIGHRALLHA-CTLESNCFVGMGAIVMDGAV----VES

DE_gi|197121924 ------------THPTVIGEDVTIGHRAVLHG-CTVHDRCLIGIGAIVLDGAV----VGP

AL_gi|83310891| --------------GTYIGSDITIGHGAILHA-CTLEDACFVGMGAVVLDGVV----VES

AL_gi|57239548| --------------NTNIGKKVTIGHGCILHA-CEIQDYVLVGMGSIIMDNVV----VEK

AcCa1 HSILGAGSVLPAWQRIPSGQ-IWVGNPAKYLRDLTEEEFDFLEKSSAHYTVLSKQHAYEF

AcCa2 HAIVGPGAVVTSGKRVPSGQ-VWAGNPAKYVRDVS-EEEKEFAGWAEKRYTQAKAHLAQT

CA1 HGMVAAGALVRQNTRIPSGE-VWGGNPARFLRKLTDEEIAFISQSATNYSNLAQAHAAEN

CA2 HAMVAAGSLVKQNTRIPSGE-VWGGNPAKFMRKLTDEEIVYISQSAKNYINLAQIHASEN

CA3 HAMVASGALVRQNTRIPSGE-VWGGNPAKFLRKVTEEERVFFSSSAVEYSNLAQAHATEN

CAL1 RSILEAGSVVPPGRRIPSGE-LWGGNPARFIRTLTNEET-LEIPKLAVAINHLSGDYFSE

CAL2 RSILEAGSVLPPGRRIPSGE-LWGGNPARFIRTLTNEET-LEIPKLAVAINHLSGDYFSE

Cre1 GSIVAAGAVVPPNTTIPSGQ-VWAGSPAKFLRHLEPEEASFIGKSASCYAELSAIHKFEQ

Cre2 GAVIAAGSYVEEGTTVPSGE-VWAGSPAKKLRDVRAGEAEYLKSLPG-----RYTELAGE

Cre3 ESILAPNSVVPPARRIPSGE-LWGGSPAKFIRKLTDHERDRVLDDVSTHYHNLATMFRRE

Ddi1 NSILGSNSILTKGSRIKSGE-LWVGKPAKFVRNLTENE-KIDISNSAHSYMLNAEKAFES

Ddi2 NGFLEAGSLLTAGKTIKSGE-YWGGSPAKFIRQVTKDDESQLEKIIEQNINLSEQHEKQT

Iga KAILEAGSVLPSGARVPSGE-VYAGNPAAFVRKLEKEEM-AAIETKADEIFELSRKHADE

Hve NSMLGANSVLLSHDRVSTGE-FWAGNP---------------------------------

Cpa1 HAIVAAGAVVPPGKTVPSGE-LWAGNPAKFLRTVTDAEKAFFTKSATEYTKLAEDHADEW

Cpa2 NSILEPGSVVVEGQRIPEGE-LWGGNPARFVRKLSESER-ADIDVVADKVYQINQDHGEE

Ehi NVLIGANSLVTSRTVIPDNS-LVMGSPAKVVRELREKEFEYLKENIKEYDDIKQGYHLEQ

Cme GAVIGAGSVLPPGTVVGAGQ-LWLGTPARFVRLVSAEEKQQFAVQCSQYVELAKMHATEC

Gth1 GAAVGPGAVVPASTVVPAGQ-IFT---SAGLRALKADELAAIAAICGNVSKMAPVHTAEC

Gth3 GCLIGAHSLVLENTEIPDGS-LVLGSPAKVVKEVSAVMREAMKSGPLTYKHKAMEFEKDL

Gth4 GAQVGPGSIVPPGRRIPANE-VWQGRPAQYVRTLTGSDSED----LDKKLKTFVKDTELH

Gth2 GAYIAPGSVVASGTVVPKAK-LFCG--EQVLRDLTPAESARLASSVESLCFLRLEHAAEV

Sec HAMIAAGAVVPPGTRVPAGQ-LWAGNPARYLRDLTYDEIDFITKSAEEYYALSKGARATF

Ram QSMLAAGTVLLAGTRVPAGQ-LWAGNPGKYVRDLTDDEIHFIAVSADVYYASAQQITAAT

Egr GAVVGAGSVVEDDQYIPAGE-VWVGRPARYLRKTGDVDTFTAVAENDTLRSLHLAYSEYE

Tbr GSMLCAASVLQSGAVIPSGE-MWAGNPAEKVRDLTEKEQDDMIKAAKHMVLLAIEHRDSW

Mja1 NAFVAAGSVVEAGTKIPEGQ-LWAGNPAQFIRELTDREREQH------------------

Mja2 GAVVAAGAFVPANTVVKAGT-VFGGSPAKEKGVVTQEDVAAYRERHAALQKAAQKHTAEH

Tth1 GAMIAANSVVPPGRLIPAGT-LWAGNPCTFVRNLTKSELATNIDHAKKQLHLAQQHRYEY

Tth2 GSVIAPNSLVPAGRLIPSGQ-LWAGSPVRYVRDLKEEEIKLNLEQTEQNLSIG-----KT

Tth3 NAVVAAGSVVPENTQVPSNQ-IWAGSPAQYLRDITPEERQVLQEHHQECVQLARIHAEET

Oma GAMVAAGALVGTDKVVKSGE-LWAGVPARKLRDLTEAEAAFIEKSAAQYVDFGIWHKCQG

Ehu HGVLEAGSVLPAGGLVPRGE-VHGGNPAAFVRKLEKDEI-AAIEKKAEDVSMSAKKHADE

Plu HAIVEPGSVLPAGGRVPSGE-MWGGNPIAFVRKLSKEEA-ADIEKQANAVAD--------

Pin1 NTIIAAGSVVPPGRRIPSGQ-LWAGNPAKYVRDLSDDEVADIAKQASEYKSIASTHS---

Pin2 KSIITAGSIVTKGKQVPSGQ-LWSGVPARYLRDLTAEETQFMQQCSSEYAQLAEQYADEC

Ptr1 NVIIAPAAIVTPGTMIPSGE-LWAGSPAKMIRVLTEDEIAAIPKQASETAALASMHAIEH

Ptr2 HVILKPGTVVMPYQRIPSGQ-MWAGNPAAFVSELTPDEK-EDIQQQALKIFTSTKEHILE

Ngr1 NVVIAAGSLVPPGRRIPHGE-MWAGSPAKFVRKLGHHEEEQVKTDAEAYVNLAEAHSLEF

Ngr2 GAMIAPGAVVTPGTIVPGGE-IWAGVPAKKLRELTPEEQESIKKSAAELSELAQVHKQEQ

Hda HAILEPGSVLTAGKVVPSSQ-VWGGSPARFIRNATPDEVANIRSAAEFYSSLAQKHSAEV

Bho NSSLASGSVVEQGKTIPAGE-YWAGNPAKFVRKVGEHEKDD------------------M

ARCH_Msa PRSAAIGVTIPDGRYIPAGMVVTSQAEADKLPEVTDDYAYSHTNEAVVYVNVHLAEGYKE

AL_gi|88607111| GSMLAAGSLLTRGKIVKSGE-LWAGRPAKFLRMMTEEEILYLQKSAENYIALSRGYL---

AL_gi|73667471| NTMLAAGSLVTKGKVIKSGE-LWAGRPAKFFRMLSEEELNHIKESADNYIRLSQEYLECR

AL_gi|83593755| WAMVAAGALVTPGKRVESRS-LWAGSPAARKRDLSAEDIAFFPESARKYADLADIYVEEM

DE_gi|197121924 DAMVGAGALVPPGAVVPPGT-LVMGQPAKPKRPLTPEEIAFLRTSAANYVSYAARYRAEG

AL_gi|83310891| GAMVAAGAVVTPGKRVKAGE-LWGGNPAKLLRRLSDEEIAFFPVSAEKYVELAAKYFKA-

AL_gi|57239548| NAMVAAGSLIVRGKVVKTGE-LWAGRPAQFLRMLSSDEIEEISKSADNYIELASDYITGK

AcCa1 Y---LPGHAYIDAEKKGIQVGYQVEPLSGEESVLLAPNYKEKVSVH--------------

AcCa2 IKLAEEKEVDLLTEDILR-EMRPGTRFAD-------------------------------

CA1 AKPLNVIEFEKVLRKKHALKDEEYDSMLGIVRETPPELNLPNNILPDKE--TKRPSNVN-

CA2 SKSFEQIEVERALRKKYARKDEDYDSMLGITRETPPELILPDNVLPGGKPVAKVPSTQYF

CA3 AKNLDEAEFKKLLNKKNAR-DTEYDSVL-------DDLTLPENVPKAA------------

CAL1 F-LPYSTVYLEVEKFKKSLG-------IAV------------------------------

CAL2 F-LPYSTIYLEVEKFKKSLG-------IAI------------------------------

Cre1 SKTFEEQYTESCIIKDRAALADPSNSVHQMWEYDSQTALVARAKR---------------

Cre2 HKGIMKVLKMKQAEYFA-------------------------------------------

Cre3 A-LEPGTAWRDVEAWRQKLVDQGEYEWINFREQKYLMR-LQHEAEALEKLTH--------

Ddi1 FGLDKDSFIYIDAQQQGIQVGWKGSYFSE-------------------------------

Ddi2 SKSAKELNNDLLQKYVK--NRTRSDHILNNPL----------------------------

Iga F-LSY-------------------------------------------------------

Hve ------------------------------------------------------------

Cpa1 KKTHGAARGG--------------------------------------------------

Cpa2 FYLPYSTAYLEL------------------------------------------------

Ehi PQNQ--------------------------------------------------------

Cme GKTPDQLDAEQMAALLWEERSEDYLSSLGLLGKEEDVMAAQKAYLAHERQLAASGAPKGS

Gth1 NKSFKDIEKEKEGYEWQQP--LEDGDPMGLLSQDHEKSLQWQSKFT--------------

Gth3 KEVKD-------------------------------------------------------

Gth4 IDCNYGIGEEHN-------MLHRDAMEAMQKGN---------------------------

Gth2 YKDVIDIEQDKSDAKWMEERSLDYDSSLGLLRTMRGHRFGKHCGRGRPDG----------

Sec APG---SLNYVQVEKIRARIGEPPLMQPSENPCAN-------------------------

Ram AVN---SLAYVE------------------------------------------------

Egr TTHGNVWAESDKVC----------------------------------------------

Tbr ELTWEELEDQREAREQFARYAENNREVRTKPMYIKEPPRPSRKAMSRKTPQEMVDGGEHK

Mja1 ------------------------------------------------------------

Mja2 AKTGSQYAQE--------------------------------------------------

Tth1 LPYNSAYLQKSNSEEDLNPTKYDDVTINYNFGDEERAQENPLKY----------------

Tth2 HKSSLIQQEAYDRLLA--------------------------------------------

Tth3 EKSFREVLNDFDRITAEAEYDHESLALQKMRDLGFPMEGEEEEYIEQRVFMREQLPPLES

Oma KTLSRFPP----------------------------------------------------

Ehu F-LAYSNTYQLREQLGTAAGKI--------------------------------------

Plu ------------------------------------------------------------

Pin1 ----------------------------------DEFLPYGTAYLDAEKIKAAGGHL---

Pin2 AKTFEEY--EADTERYKILRDVGETGLPQK--GDERED--TGLYFRY-------------

Ptr1 SKSYEQVMEEEQVAENELYREVPVPKQTENPLGDVLGQGMPGRIFRSTLSHPEDIYKGQQ

Ptr2 F-LPYGRTFVHLEELEKQAGLQVKQG----------------------------------

Ngr1 TSFGKAYKEVDVIADKLEQINPESVDGRPVHWQVWHEQSKNATVALWKKDRIL-------

Ngr2 DKEFEELLHDMETFKFREDRLEEYTYEKVESPSSTAPTKN--------------------

Hda SKTEGEREMERVRDEVSPSNPYPEENLESIPQNTKSSSPKH-------------------

Bho LQYSRDCTE---------------------------------------------------

ARCH_Msa TS----------------------------------------------------------

AL_gi|88607111| ------------------------------------------------------------

AL_gi|73667471| ------------------------------------------------------------

AL_gi|83593755| ------------------------------------------------------------

DE_gi|197121924 GVP---------------------------------------------------------

AL_gi|83310891| ------------------------------------------------------------

AL_gi|57239548| L-----------------------------------------------------------

AcCa1 ------------------------------------------------------------

AcCa2 ------------------------------------------------------------

CA1 ------------------------------------------------------------

CA2 ------------------------------------------------------------

CA3 ------------------------------------------------------------

CAL1 ------------------------------------------------------------

CAL2 ------------------------------------------------------------

Cre1 ------------------------------------------------------------

Cre2 ------------------------------------------------------------

Cre3 ------------------------------------------------------------

Ddi1 ------------------------------------------------------------

Ddi2 ------------------------------------------------------------

Iga ------------------------------------------------------------

Hve ------------------------------------------------------------

Cpa1 ------------------------------------------------------------

Cpa2 ------------------------------------------------------------

Ehi ------------------------------------------------------------

Cme GKPETKISSSTDARQQVSAGH---------------------------------------

Gth1 ------------------------------------------------------------

Gth3 ------------------------------------------------------------

Gth4 ------------------------------------------------------------

Gth2 ------------------------------------------------------------

Sec ------------------------------------------------------------

Ram ------------------------------------------------------------

Egr ------------------------------------------------------------

Tbr PPLAESIQQGY-------------------------------------------------

Mja1 ------------------------------------------------------------

Mja2 ------------------------------------------------------------

Tth1 ------------------------------------------------------------

Tth2 ------------------------------------------------------------

Tth3 EFWKKNYDPYEQDLFHFPDSFKAYQQQYKRYDEAKKYFEENPNVEATIIDREFKEPTNKK

Oma ------------------------------------------------------------

Ehu ------------------------------------------------------------

Plu ------------------------------------------------------------

Pin1 ------------------------------------------------------------

Pin2 ------------------------------------------------------------

Ptr1 PK----------------------------------------------------------

Ptr2 ------------------------------------------------------------

Ngr1 ------------------------------------------------------------

Ngr2 ------------------------------------------------------------

Hda ------------------------------------------------------------

Bho ------------------------------------------------------------

ARCH_Msa ------------------------------------------------------------

AL_gi|88607111| ------------------------------------------------------------

AL_gi|73667471| ------------------------------------------------------------

AL_gi|83593755| ------------------------------------------------------------

DE_gi|197121924 ------------------------------------------------------------

AL_gi|83310891| ------------------------------------------------------------

AL_gi|57239548| ------------------------------------------------------------

AcCa1 ------

AcCa2 ------

CA1 ------

CA2 ------

CA3 ------

CAL1 ------

CAL2 ------

Cre1 ------

Cre2 ------

Cre3 ------

Ddi1 ------

Ddi2 ------

Iga ------

Hve ------

Cpa1 ------

Cpa2 ------

Ehi ------

Cme ------

Gth1 ------

Gth3 ------

Gth4 ------

Gth2 ------

Sec ------

Ram ------

Egr ------

Tbr ------

Mja1 ------

Mja2 ------

Tth1 ------

Tth2 ------

Tth3 PWTRKY

Oma ------

Ehu ------

Plu ------

Pin1 ------

Pin2 ------

Ptr1 ------

Ptr2 ------

Ngr1 ------

Ngr2 ------

Hda ------

Bho ------

ARCH_Msa ------

AL_gi|88607111| ------

AL_gi|73667471| ------

AL_gi|83593755| ------

DE_gi|197121924 ------

AL_gi|83310891| ------

AL_gi|57239548| ------
